# Mechanochemical Forces as a Synthetic Tool for Zero- and One-Dimensional Titanium Oxide-Based Nano-photocatalysts

**DOI:** 10.1007/s41061-019-0262-3

**Published:** 2019-11-25

**Authors:** Dimitrios A. Giannakoudakis, Gregory Chatel, Juan Carlos Colmenares

**Affiliations:** 1 Institute of Physical Chemistry, Polish Academy of Sciences, Kasprzaka 44/52, 01-224 Warsaw, Poland; 2 Université Savoie Mont Blanc, LCME, 73000 Chambéry, France

**Keywords:** Mechanochemistry, Sonochemistry, Ultrasound-assisted synthesis, One-dimensional titanium oxide, Photocatalysis, Ball milling

## Abstract

**Abstract:**

A new field where the utilization of mechanochemistry can create new opportunities is materials chemistry, and, more interestingly, the synthesis of novel nanomaterials. Ball-milling procedures and ultrasonic techniques can be regarded as the most important mechanochemical synthetic tools, since they can act as attractive alternatives to the conventional methods. It is also feasible for the utilization of mechanochemical forces to act synergistically with the conventional synthesis (as a pre-treatment step, or simultaneously during the synthesis) in order to improve the synthetic process and/or the material’s desired features. The usage of ultrasound irradiation or ball-milling treatment is found to play a crucial role in controlling and enhancing the structural, morphological, optical, and surface chemistry features that are important for heterogeneous photocatalytic practices. The focus of this article is to collect all the available examples in which the utilization of sonochemistry or ball milling had unique effects as a synthesis tool towards zero- or one-dimensional nanostructures of a semiconductor which is assumed as a benchmark in photocatalysis, titanium dioxide.

**Graphical Abstract:**

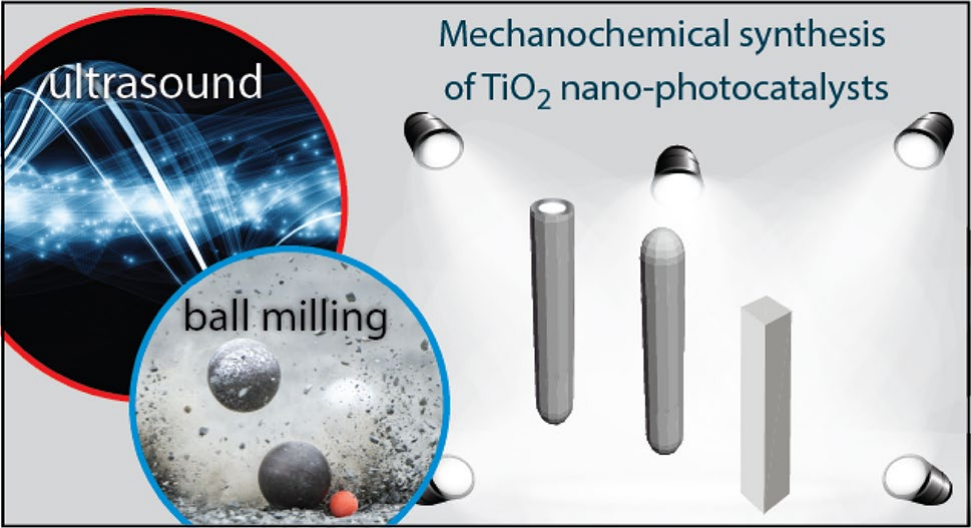

## Introduction

### Nanotechnology and Photocatalysis

Generally, the impact of nanotechnology has been astonishingly positive in the last decades toward a wide range of environmental, energy, catalysis, as well as synthetic applications and technologies, with a continuous incremental trend of published peer-reviewed articles. Nanostructured and nanoengineered materials garner continuously enhanced research attention and focus due to their unique and novel properties, especially in comparison to bulk materials/counterparts. Application of nanoscaled materials covers a broad range of fields, from electronics and catalytic reactions, to medical and environmental remediation, while novel nanomaterials for new applications are highly desired. The properties of nanomaterials depend on the morphological (shape and size), structural (surface area and porosity), optical (bandgap and light-harvesting capability), and surface chemistry features (nature and availability of the reactive sites). Another important aspect is to obtain well-defined phase composition of high homogeneity. All the above features are directly linked and dependent on the method/protocol of preparation [1]. By tuning specific synthetic parameters, such as temperature, aging, and washing protocol, it is feasible to control the vital features for a targeted use. For instance, the chemical composition and the porosity are crucial features regarding catalytic synthesis and environmental applications. On the other hand, the optical and morphological features are more important for fabricating crystals for photonic devices.

Another important target in laboratory as well as in industrial research is to find novel ways to conduct reactions following “green” approaches. And toward this direction, photocatalysis is a favorable tool, since it is feasible to utilize a natural source of energy, solar light. The harvesting of light from a photocatalyst can promote specific reactions even without the use of additive chemicals or another source of energy. The most important part in heterogeneous photocatalysis is the development and usage of materials that can function as sufficient photocatalysts, and nanotechnology has been shown capable of providing solutions. Synthesis of nanomaterials and tuning specific features of them like nano-morphological and optical features is an ultimately important and efficient strategy to achieve the above. Even though the synthesis of nanoscaled photocatalysts has been a hot topic during the last decades with many published articles and end-use applications, the use of mechanochemical-based synthetic approaches is not so broadly explored. By gathering the existing knowledge on the effects derived from the mechanochemical forces like ultrasound (US) irradiation and ball milling, it will be realistic to go a step further. The focus of this work is to collect all the reports in which the two above-mentioned techniques were applied during the synthesis of two benchmark semiconductor photocatalysts, titanium dioxide or titanate, in order to obtain various polymorphs with different structural, morphological, and optical features.

### Mechanochemical Synthesis

The exploration and discovery of new synthetic approaches as well as the incorporation of advantageous techniques for the development of new or improved properties of already known nanomaterials as photocatalysts is an ongoing and interesting field of research, with fascinating potential [1, 2]. In recent years, mechanochemical processes were found to hold great promise, since they are effective and can lead to nanomaterials of novel properties. Another important aspect is that various reported active nanocatalysts can be synthesized in a shorter time compared to traditional wet-chemistry synthesis. In many cases, the design of mechanochemical-based methods can have a positive effect on the “green” character and environmental footprint: consumption of less energy, less or even no use of ﻿hazardous solvents, need of recycling, purification, etc. According to the International Union of Pure and Applied Chemistry (IUPAC), the definition of a mechanochemical process is: “*a chemical reaction that is induced by the direct absorption of mechanical energy*” [3]. The utilization of mechanochemical forces holds great promise and begets novel approaches in nanocrystalline synthesis (mechanosynthesis), and, more specifically, on how to control the desired features, crucial for different applications [3–7]. Herein, two mechanochemical sources will be introduced: (1) US irradiation (sonochemistry) and (2) ball milling. The rapid growth of the research interest around the utilization of mechanochemistry methods is due to their unique effects. By the correct selection of these effects, it is feasible to obtain novel nanomaterials, and to control desired physical, chemical, and optical properties [5, 8]. Simultaneously, it is possible to eliminate the environmental footprint of the synthesis, avoiding, for instance, the usage of high energy, hazardous and non-recyclable chemicals, or by decreasing the duration and the number of steps of the synthesis [9].

### Sonochemistry

#### A Brief History

Sound waves not detectable by the human ear with frequencies ranging from 20 kHz to 200 MHz are referred to as ultrasound (US) waves [10]. The effects of sonication are linked to the cavitation phenomena, and they can be chemical, physical, mechanical, or optical. The first reference to the cavitation phenomena by Thornycroft and Barnaby dates from 1895 [11]. By the time Neppiras introduced the term “sonochemistry” in 1980 [12] and Makino et al. showed the formation of radical species during the sonolysis of water in 1982 [13, 14], the research attraction of sonochemistry had increased dramatically. In general, sonochemistry is linked with the understanding and interpretation of the processes and the effects initiated by US irradiation due to the cavitation phenomena. The main derived results are the enhancement of the reaction rate, radical species formation, as well as mass and heat transfer [1, 2, 4, 15–17].

#### Cavitation Phenomena—Mechanistic Aspects on “How Does Everything Work?”

The formation of cavitation bubbles is due to pressure changes upon the travel of US waves in a liquid. The initially formed microbubbles, consisting of vaporized solvent or/and dissolved gases, grow continuously in size by absorbing energy during the irradiation [18]. After growing to a certain size, they violently collapse, creating a localized “hot spot.” The local pressure and temperature can be above 1000 bars and 5000 K, respectively [18–20]. The hot-spot concept and the consequential effects can be described by distinguishing three zones [21, 22]. Zone 1 is inside the bubble (primary sonochemistry), zone 2 is at the gas–liquid interface (secondary sonochemistry), and zone 3 is the bulk liquid phase surrounding zone 2. At the interior of the cavity, cleavage of bonds and formation of radicals occurs due to the harsh energetical environment and to the fact that the gaseous concentration is extended [22–24]. These radical species can also be transported to zone 2, where reaction of free radicals and pyrolysis can take place, or even to the bulk liquid zone 3. In an aqueous environment, the sonolysis of water can lead to the formation of hydroxyl (HO^·^) or hydroperoxyl (HO_2_^·^) radicals, and hydrogen peroxide (H_2_O_2_). These species can initiate secondary reactions that can play a key role in material synthesis or for catalytic reactions [15].

Even if the “hot-spot” theory is the most accepted to explain these phenomenas, several studies lead to the proposition of plasma [25, 26], electrical [27], and supercritical water [28] theories, demonstrating that all the mechanisms involving US are not completely known. In addition to the chemical effect of US, various other mechanisms exist, such as physical, mechanical, or light emission (sonoluminescence). The latter one is also valuable in order to determine the active regions and intensity of the US waves using a hydroxyl radical trapping agent, luminol (through chemiluminescence [29–31]. The physical/mechanical effects can be the formation of microjets, turbulence, microstreaming, shockwaves, and agitations [23]. These effects can positively promote the reaction rates by affecting the mass (mixing) and heat transfer phenomena or resulting in some structural alterations of the solids, such as erosion, exfoliation, fragmentation, or deformation [6, 8, 22, 32, 33].

The utilization of US irradiation is a complex aspect, since the formation of cavitation in liquids can be affected by numerous parameters [2, 22, 23]. Some of them are described in most of the articles, but some were not reported. The frequency and the power of the irradiated US waves can be considered the most fundamental parameters [16]. Increase of the US frequency leads to shortening of the expansion and compression pressure cycle, and, as a result, to a negative impact on the effectiveness. The formed bubbles/cavitation at higher frequencies have a smaller size and less violent implosion effects, although they have a better size distribution and rate formation. At lower frequency, the cavitation phenomena is more violent and intense with a consequent of higher localized pressure and temperature, as well as higher concentration of free radical formation. However, there are many cases where the high frequencies have desired impact on reaction rates and material synthesis.

Other important parameters that should be taken into consideration for the effective US utilization in synthesis of catalysts and catalytic reactions are the solvent, the presence/concentration of dissolved gases, temperature, and pressure [34]. The physicochemical parameters of the solvent, for example, the solubility of air or oxygen, viscosity, surface tension, or vapor pressure, play key roles in the cavitation threshold. The increment of the latter parameter has a negative impact on the cycle formation, while, on the contrary, increment of the rest has a positive effect. Initiation of cavitation is facilitated by the presence of dissolved gases. However, the extent of the assistance upon cavitation is related to the physical properties of the gas. Contrary to the chemical processes, the increase of the temperature (until a specific range) has a negative impact on the sonochemical reaction due to increase of the vapor pressure and to the decrement of the gaseous solubility. However, there are many circumstances revealing that the temperature increase has positive and desired effects. An increase of the reactor pressure could cause a decrease of the solvent’s vapor pressure.

### Ball Milling

#### A Brief History

﻿The earliest recorded mechanochemical process, according to Takacs [35], dates to the fourth century BC, in which Theophastus of Eresos noted the synthesis of elemental mercury by grinding cinnabar (HgS) with acetic acid in a Cu vessel, the first documented separation of an elemental metal [3, 7, 35]. Since a solvent was needed even in a minimal amount, this process is regarded nowadays as liquid-assisted grinding (LAG). From this point and afterward, mechanochemistry-based approaches were applied widely in metallurgy and mining, and more details can be found elsewhere [35–37]. By the use of a pestle and mortar and without a liquid (dry grinding), it was the great experimental physicist, Michael Faraday (discovered the laws of electrolysis, electromagnetic induction, and the rotation of polarized light by magnetism) who conducted displacement reactions between a metal and oxides of less reactive metals [3, 7, 35]. In his first research published in 1820, which can be assumed as the first systematic study of the mechanochemical process, he showed the oxidation of Zn by the reduction of AgCl to Ag by simple mortar grinding [35, 38]. However, there was no specific evidence indicating if the grinding promoted the reaction mechanochemically of just thermodynamically by heat generation through friction.

**Fig. 1:**
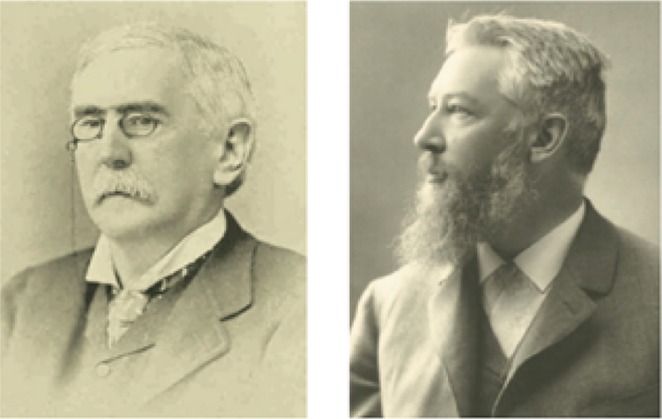
Mathew Carey Lea (1823–1897) and Friedrich Wilhelm Ostwald (1853–1932) left and right, respectively. Reprinted with permission from [40]. Copyright (2013) Royal Society of Chemistry

The American chemist and pioneer of photographic chemistry, Mathew Carey Lea (1823–1897), systematically studied and determined (between 1889 and 1894) that the above kind of redox reactions were initiated/activated by mechanochemical effects rather than thermochemical effects. A clear and loud example was the formation of elemental Ag from grinding of silver halides, while it was just melted without decomposition upon thermal treatment [39] (Fig. 1).

The terminology of mechanochemistry was introduced by L. Crismer in 1912 in a biography of Walthere Spring and his geology-oriented research on the effects derived by high pressure on powdered materials in order to explain the formation of various natural minerals [35, 41]. Although mechanochemistry started to be assumed and accepted as a distinct/separate subdiscipline of chemistry in 1919, a physical chemist and philosopher, the Nobel Laureate Friedrich Wilhelm Ostwald (Nobel prize in Chemistry, 1909, “in recognition of his work on catalysis and for his investigations into the fundamental principles governing chemical equilibria and rates of reaction”) introduced it as a separate chemistry sub-discipline alongside electrochemistry, photochemistry, and thermochemistry [42]. Ostwald made this classification due to the fact that different types of energy are required in each sub-discipline.

#### Mechanistic Aspects on “How Ball Milling Works?”

Even nowadays, there is not a complete and comprehensive mechanistic picture regarding how ball milling and, in general, mechanochemistry works. This is also linked with the diversity of the utilized techniques/equipment (like mortar/pestle, mixer and planetary mills, glass vessel or tube disperser milling, etc. [3]) and the reaction types (dry or wet), conditions (gaseous atmosphere, temperature, etc.), and precursors (minerals, metal oxides, metals, chemicals, etc.). Several processes take place during the ball milling like heat and mass transfer.

**Fig. 2:**
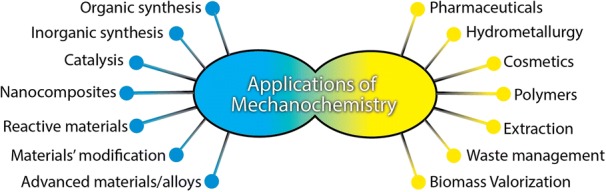
Applications of mechanochemistry

However, the most crucial driving force is believed to be the generation and relaxation of mechanical stress that have a direct effect on the crystalline lattices [3]. The theory of “hot spots” formation during ball milling is a widely accepted one [7]. Hot spots can be created by the cracking of crystals, resulting in local temperature (up to 5000 K) and pressure, electric fields up to 108 V/m, crack propagation with velocity close to that of sound (105 cm/s), and lifetimes for bond excitation around 100 fs [3, 43, 44]. These effects are analogues of those of US irradiation in a liquid, even though the formation of a hot spot results from the cavitation phenomena. And it is important that the high amount of added energy is localized microscopically, even nanoscopically, without affecting the macroscopic system to a great extent. This localized high amount of energy can lead to a diversity of consequences, such as lattice deformation, cleavage of bonds, or formation of radicals. And these phenomena cannot be achieved by other synthetic approaches in solution. Figure 2 presents the most important fields of mechanochemistry applications based on the report by Elena Boldyreva [43]. More mechanistic aspects and fields of application of mechanochemistry can also be found elsewhere [3, 7, 35, 44]. Even though Boldyreva did not consider US irradiation in her work, the latter can be utilized for the same fields and applications when a liquid phase is required. The rapid growth of the research interest around the application of mechanochemistry is due recent discovery of unique effects. By the correct utilization of these effects, it is feasible to obtain the desired nanostructured materials and enhance their crucial features by simultaneously eliminating the environmental footprint of the synthesis and avoiding the usage of high energy and hazardous and non-recyclable chemicals.

### TiO_2_: The Benchmark Semiconductor Photocatalyst

Titanium dioxide (TiO_2_) can be regarded as one of the most popular semiconductor photocatalysts, for a wide range of applications; organic pollutant degradation, hydrogen production, solar cells, photocatalysis, etc. It combines high photo-activity for various reactions, high stability, low cost, and low toxicity for humans, animals, and the environment. Use of titanium dioxide started intensively in 1972, when Fujishima and Honda revealed photocatalytic water splitting by titania electrodes [45]. Since then, numerous articles have focused on the use of TiO_2_ and its composites for green-oriented heterogeneous catalysis, like valorization of biomass and upgrading of obtained chemicals [1, 46, 47].

**Fig. 3:**
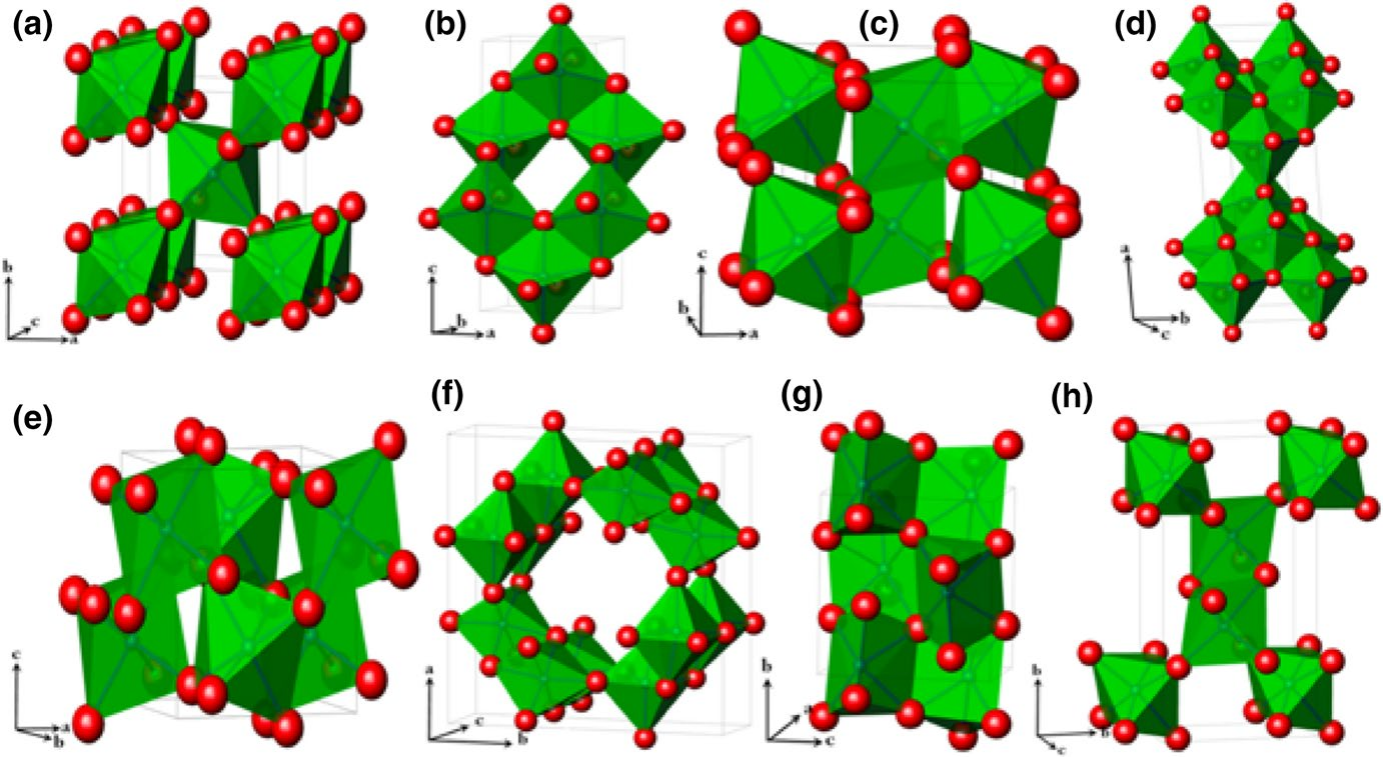
Crystal structures of rutile (**a**), anatase (**b**), bronze (**c**), brookite (**d**), columbite (**e**), hollandite (**f**), baddeleyite (**g**), and ramsdellite (**h**) phases. Reprinted with permission from [49]. Copyright (2015) Elsevier

Another important property of titanium dioxide is its superhydrophilicity that is crucial for solar fuel production and environmental remediation applications [48]. However, one crucial drawback arises due to the fact that TiO_2_ has a wide bandgap ranging from 3.1 to 3.7 eV, and UV light irradiation is required in order to trigger the photoreactivity. Considering that solar light consists predominately of ﻿visible and infrared light, with ultraviolet light less than 5% of the total solar light, a persistent research effort is focused on narrowing of the bandgap and, as a result, increase of light absorption and photoreactivity under sunlight. Even though several polymorphs/crystal structures of TiO_2_ exist [49], with the most important presented in Fig. 3, only a few of them have been studied and found promising for photocatalytic applications like biomass valorization [33, 50, 51]. The three most studied and stable crystalline phases of titanium oxide are anatase, brookite, and rutile, with the former one possessing the highest photocatalytic activity and the latter the highest stability [52]. Among the various commercially available forms of TiO_2_, one of the most active and widely studied is Degussa P25, and, in many cases, it acts as a benchmark (industry standard) [53].

There are many reported methods for the synthesis of nanostructured TiO_2_ materials. The sol–gel method is the most often applied method, but, unfortunately, it leads to amorphous nanomaterials, and, so, further treatment is needed to induce crystallization, like annealing. On the other hand, hydrothermal-based methods can promote the crystallinity and shape morphology formation, and can be used for larger scaled synthesis compared to the sol–gel method. Crystallinity in relation with the particle size is found to determine the photo-reactivity not only in the case of Ti-based catalyst [48, 54–57], but also for other materials like ZnO [58–60], MnO_2_ [61], graphitic carbon nitride [5, 62–65], or other metal oxides/hydroxides [66–68]. However, the control of the final material’s morphological features is related to a wide range of parameters during the synthesis.

Another important aspect in photocatalysis is the rate of the surface reactions. The structural (surface area and porosity) as well as the morphological features (shape and size) play a key role in the catalysis rates, and, as a result, the engineering of these features is also important [47, 69–80]. Toward the above-mentioned direction, different strategies/approaches were followed for the nano-engineering of the TiO_2_ toward key features/properties for application in catalysis. The most important strategies in order to positively trigger the photoreactivity are the following: (1) controlling the crystallographic nature; (2) introducing Ti^3+^ species and ﻿lattice disorder; (3) doping with metal or non-metal; (4) decreasing the size of the particles to nanoscale; (5) ﻿engineering the shape to 0D, 1D, 2D, 3D, or amorphous; (6) decreasing the size of the particles; (7) chemical modification like hydroxylation/hydrogenation; (8) porosity enhancement; (9) narrowing the bandgap toward the visible range of light; (10) enhancing light absorption; and (11) limitation of e^−^/h^+^ recombination.

### Our Approach to Organize this Article

The focus of the work herein is based on novel mechanochemical-assisted synthesis/modifications, such as US irradiation and ball milling, in which their utilization has led to beneficial effects in the enhancement of photocatalytic capability. Since the application of mechanochemistry has only lately been assumed and recognized as a useful process-intensification tool, in most works where US or ball milling were applied, the reports have predominately a materials point of view approach without studying a potential photocatalytic reaction. Additionally, the one-dimensional-inspired spatially ordered nanotubular-shaped titanate has gathered intense attention upon its discovery in 1995 [81]. Although the utilization of mechanochemistry in order to improve the synthesis and control specific features has been explored, the photocatalytic capabilities of these titanate nanotubes (TiNTBs) was studied in only a few of these reports. We believe that TiNTBs can display important photocatalyst behavior, and we actively work towards this direction. Based on the above and the available articles, we organized this article into two main parts/sections. In the first part, we collected the reports in which sonication was used in order to obtain nano-engineered materials with enhanced specific features for photocatalytic application, like light absorptivity, decreased bandgap, defects like surface oxygen vacancies, hydroxylation, porosity, etc. The second part is focused on the ball milling-based synthesis/modification approach. Each part is separated in two subsections. The first subsection is focused on zero-dimensional (0-D) photocatalysts, with an emphasis on how to promote the most vital of photochemistry features. In the second subsection, we collect all the research on the synthesis of one-dimensional (1-D) nanostructured titanate, like nanotubes (NTBs) and nanorods.

## PART A—Sonication-Assisted Approaches

### 0-D Particles

#### Increasing the Porosity

The supramolecular assembly sol–gel method using surfactant molecules as a template/structure-directing agent for the synthesis of mesoporous titania was reported firstly in 1995 by Antonelli and Ying [82]. The main drawback of the obtained hexagonally packed mesostructured TiO_2_ was the presence of residual phosphorous from the alkyl phosphate surfactant. From then, different long-chain organic molecules were studied as phosphorus-free surfactants. In 2000, Wang et al. [83] reported a novel synthesis of mesoporous nanostructured titanate of a high porosity, by simultaneous ultrasonication during the synthesis (1.13 cm in diameter Ti horn, 20 kHz, 100 W/cm^2^). An ethanolic solution of the organic amine and titanium isopropoxide was added slowly to a doubly distilled water, followed by aging for 6 h. The addition and the aging occurred under high-intensity ultrasonication, with the maximum temperature reaching 80 °C. The removal of the surfactant from the obtained powder by centrifugation was achieved by dilute ethanolic HNO_3_ solution and washing with ethanol. The dried powder was also calcinated in a vacuum at 350 °C (8 h) or 450 °C (4 h). Three different long-chain organic amines (decylamine, dodecylamine, and octadecylamine) were studied as the structure-directing agents.

**Fig. 4:**
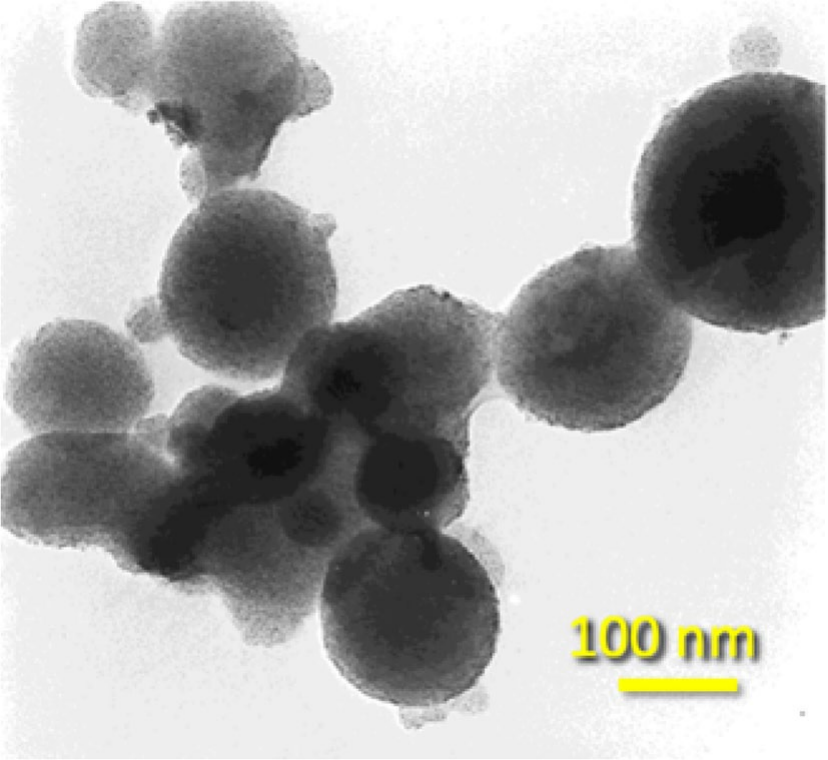
HRTEM images of the as sono-chemically prepared mesoporous titanium oxide with wormhole-like framework structures. Reprinted with permission from [83]. Copyright (2000) Wiley

The result was spherical or globular particles between 50 and 200 nm as an aggregation of very small nanoparticles, as can be seen from the high-resolution transmission electron microscopy (HRTEM) image (Fig. 4). The X-ray diffraction (XRD) analysis showed an amorphous nature even after calcination at 350 °C, but the rise of the calcination temperature to 450 °C led to an anatase crystallinity. The surface areas after extraction, and calcination at 350 °C and 450 °C, were 853, 467, and 79 m^2^/g, respectively. These values are high for metal oxides and higher than analogous titanate hexagonal mesoporous framework structures synthesized by hydrothermal and then thermal treatment with dodecylamine as a structure-directing agent (710 m^2^/g) [83, 84]. The most interesting outcome was the that the obtained nanoparticles had a structure of disordered wormhole framework, rather than a long-ranged hexagonal structure. This kind of channel motif and the high surface area are ultimately important for catalytic application, due to the improved diffusion and the availability of the active reaction sites. Compared to various other reports for the synthesis of mesoporous TiO_2_ nanoparticles, the benefits of this sonochemical method is the simplicity and rapid rate of synthesis, that leads also to nanoparticles of a high porosity. The authors linked the role of US irradiation to the accelerated condensation/polymerization of titanium hydroxide at the interface of the gas phase of the hotspots and the bulk solution.

#### Controlling the Crystallinity

In 2000, Huang et al. studied the role of US irradiation in the selective synthesis of anatase or rutile phases from different precursors and conditions [52]. The synthetic protocol was based on the addition of different precursors (TPT: tetraisopropyltitanate, TTC: titanium tetrachloride, or a mixture of TPT and TTC) in water under sonication by a directly immersed horn (20 kHz, 100 W/cm^2^). The suspension was ultrasonic-aged for 3 h, with the temperature reaching 80 °C. The precipitates were obtained by centrifugation and subsequently washed with deionized water and ethanol, following by overnight vacuum drying. Compared to the sol–gel-derived materials that were amorphous prior to calcination, the samples obtained via US irradiation showed a high degree of crystallinity. Rutile-phased nanoparticles (crystallographic size based on the application of Scherrer's formula at the XRD: 8.2 nm) were obtained when TTC was used, and anatase phase (3.5 nm) in the case of TPT. The materials synthesized via US were also found to have relatively high surface areas, 103 m^2^/g for the rutile phase and 201 m^2^/g for the anatase phase. The TPT-derived sample (anatase) had a broad size distribution of mesopores (average of 5 nm), linked by the authors to the aggregation of nanoparticles. The rutile phase TTC-derived sample had a non-mesoporous nature.

When a mixture of TPT and TTC were used in a molar ratio of 63.4:36.6, a mixed anatase/rutile phase was determined. Analysis of the powder X-ray diffraction (PXRD) data revealed a crystallographic ratio of anatase to rutile phases of 47.6:52.4, suggesting that in the case of the mixture, part of the rutile phase formed at the expense of TPT. It should be pointed out that without US irradiation, the material obtained with the same mixture of precursors as above mixture was amorphous. The authors also studied the role of temperature. When the synthesis was conducted at 30 °C instead of 80 °C and TPT as precursor, the result was mixed brookite and anatase phases. On the contrary, when TTC was hydrolyzed under sonication at 10 °C, rutile phase was obtained. The role of pH was also studied, but not in detail. When TPT was hydrolyzed under sonication at pH 0.7, the obtained sample of limited mass had a mix of rutile and anatase phases. Increasing the pH of the supernatant of the above synthesis to 8.6 and further sonication for 3 h led to a pure anatase phase. Based on all the above, it is obvious that even though US irradiation and temperature play a key role by promoting the crystallization, the pH can determine the finally crystallographic phase. Another outcome derived by the authors was that the hydrolysis of TPT in water is slower compared to TTC, resulting in a more homogeneous and partly condensed gel. The formation of a hotspot due to US waves inside the gel phase promotes the polycondensation of the Ti–OH species and the formation of a large number of seed nuclei, leading to smaller nanoparticles.

In 2001, Yu et al. [85] studied the effect of US irradiation (cleaner bath, 47 kHz, 120 W_elec._) as well as the role of the ethanol-to-water ratio during the hydrolysis upon precipitation of titanium tetraisopropoxide in pure water or mixed EtOH–H_2_O solution at different ratios, followed by in-air calcination at 500 °C for 1 h. The ratio of ethanol to water was found to play a key role in the crystallinity of the final powder, and, as a result, in the photocatalytic reactivity. While in pure aqueous solution, the obtained material had a mix of anatase and brookite phases (in a ratio of around 80:20); the addition of methanol led to the elimination of the brookite phase. The materials obtained without using methanol were found to possess a higher photoactivity against the oxidation of acetone in air compared to P25. On the contrary, the material with a solely anatase phase showed the lowest oxidative performance. The authors linked this to the fact that the presence of two crystallographic phases has a positive impact on the photocatalytic activity, by decreasing the combination of the photogenerated e^−^/h^+^ pairs. In 2010, Ghows et al. synthesized nanosized TiO_2_ by hydrolysis of titanium tetra-isopropoxide in a solution of ethanol/water under low-intensity and high-frequency (500 kHz) sonication [86], although they did not study their photocatalytic properties. The crystalline phase and particle size were dependent on the ethanol-to-water ratio, US irradiation time, and temperature.

#### Altering the Surface Chemical Features and Bandgap

In 2011, Chen et al. [87] reported that the distortion and doping of the outer surface of TiO_2_ nanoparticles by high-pressure and high-temperature hydrogenation led to an enhancement of the visible light absorption. Interestingly, and for the first time, the obtained TiO_2_ powder did not have the characteristic white color, but a deep dark one. The reported synthesis of this material was conducted in two phases. In the first phase, titanium dioxide nanocrystals of an ~8-nm diameter were synthesized by a sol–gel method, using an organic template and acid (pluronic F127). The white powder obtained after calcination (500 °C, 6 h) underwent hydrogenation under a high-pressure (20 bars) H_2_ atmosphere at ~ 200 °C for 5 days, resulting in a black powder, stable even after 1 year.

**Fig. 5:**
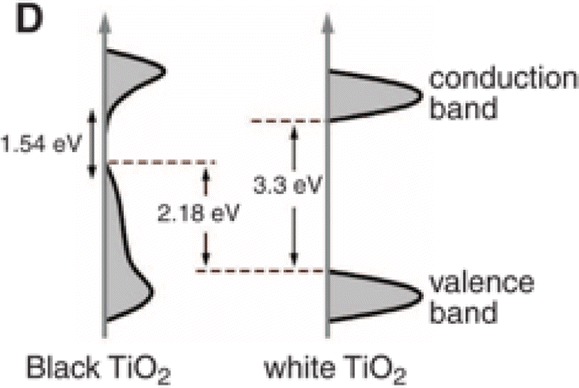
A schematic illustration of the density of states (DOS) of disorder-engineered black TiO_2_ compared to that of the white TiO_2_ precursor. Adapted with permission from [87]. Copyright (2011) American Association for the Advancement of Science

The HRTEM analysis revealed no shape alteration upon hydrogenation; however, an outer disordered layer around 1 nm in thickness appeared. The X-ray diffractogram of both white and black samples revealed the characteristic peaks of the anatase structure. The Raman spectrum of the white sample showed the six typical Raman-active modes of the anatase structure. The Raman spectrum of the black sample revealed a broadening of the six typical Raman-active modes which were appeared in the white powder, and some additional new bands not linked to any of the three classic polymorphs of TiO_2_. The X-ray photoelectron spectroscopy (XPS) analysis revealed an almost identical and impurity-free bonding environment for Ti. On the contrary, the hydrogenation also resulted in a new O 1s peak (at 530.9 eV) which was attributed by the authors to the formation of Ti–OH moieties. Since the dangling bonds tends to attract hydrogen, the authors expected that the H doping occurred predominately in the outer disordered layer where more dangling bonds exist compared to the inner crystalline core. The bandgap of the non-hydrogenated materials was determined by diffuse reflectance as 3.3 eV (slightly higher than bulk anatase). The black TiO_2_ showed a dramatically narrower bandgap , while the onset of the optical absorption started from ~ 1200 nm (1.0 eV). The authors linked this to the “band tail states” phenomena, where the valence and conduction bands narrow. ﻿The density of states (DOS) of the black sample compared to the white one can be seen in Fig. 5. The photocatalytic activity against methylene blue dye was found to be faster by ~ 7.5 folds under solar irradiation, and the photo-activity was found to be stable even after eight cycles. More interestingly, the black titania sample was found capable of photocatalytic hydrogen production from water under sunlight, with a rate two folds higher than the best semiconductor catalysts at that time. The non-hydrogenated sample was not found photoreactive for water splitting, even after loading with Pt. The H production was repeatable for more than 20 cycles. The authors showed that the hydrogenated TiO_2_ did not act as an H reservoir, since 40 mg of H_2_ were formed after 100 h of irradiation, with the sample having around 0.05 mg of hydrogen.

In 2012, Osorio-Vargas et al. studied the effect of low-frequency US irradiation (20 kHz, 1.2 W/mL) on P25 [88]. Based on electron spin resonance (ESR) measurements, they reported evidence to support the formation of oxygen vacancies for the obtained sample after 6 h of irradiation. These vacancies can be responsible for enhance visible light absorption, and also for the obtained grey-shaded color, although the photoreactivity was not studied. These surface chemistry alterations were assigned to the shock waves from the cavitation phenomena and high-velocity interparticle collisions.

**Fig. 6:**
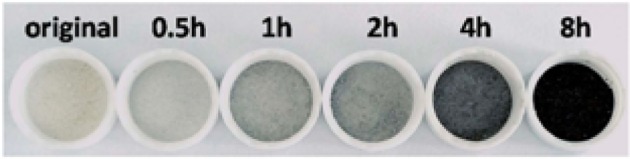
The powders obtained after different ultrasound irradiation duration. Reprinted with permission from [89]. Copyright (2015) Springer Nature

In 2015, Fan et al. utilized ultrasonication in order to synthesize amorphous and porous hydroxylated black TiO_2_ [89], avoiding the harsh and expensive synthesis by hydrogenation at high pressure (20 bars) and temperature (200 °C). The pivotal role of US waves during the synthesis was determined by varying the irradiation duration (0.5–8 h), leading to different shades of blackness. At the first step of synthesis, titanium sulfate [Ti(SO_4_)_2_] and ammonia water were inserted in aqueous phase inside an ice-water bath in order to control the reaction rate (2 h, under magnetic stirring). After centrifugation and US washing (25 kHz, 100 W, 20 min) by deionized water, the dispersion was treated with high-power US irradiation (25 kHz, 1500 W/100 mL) using a probe. The synthesis was conducted at 80 °C under different US irradiation durations; 0.5, 1, 2, 4, and 8 h. Afterward, the obtained materials were dried at 80 °C. The degree of the black shade was increased by extending the US irradiation (Fig. 6). After 8 h of US exposure, the obtained powder had a deep black color. It was pointed out that by the application of lower-intensity US irradiation, no powder with a black shade was obtained.

**Fig. 7:**
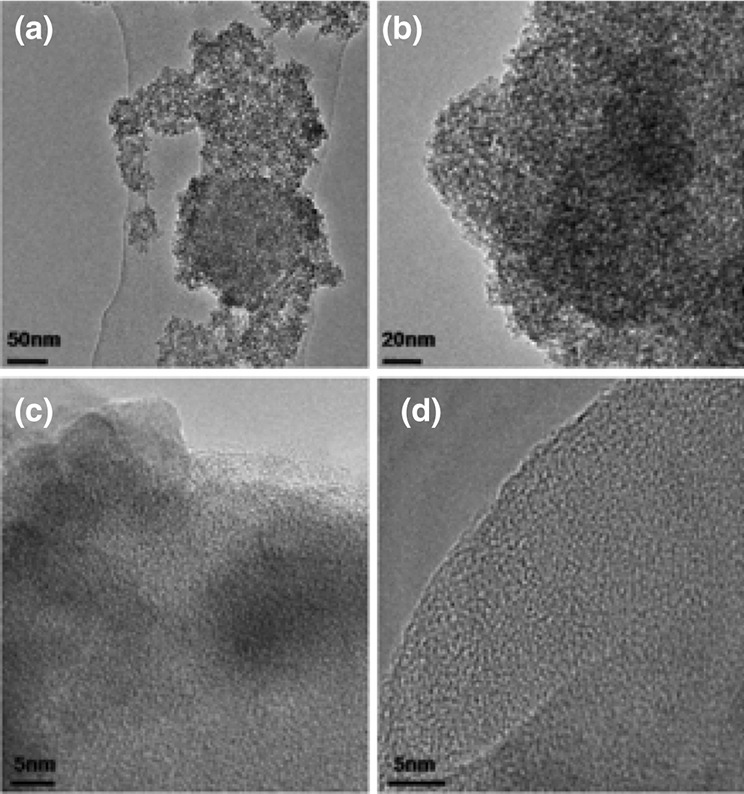
TEM and HRTEM images of non-ultrasound-treated white TiO_2_ (**a**, **c**) and amorphous hydroxylated black TiO_2_ obtained after 8 h of ultrasonication (**b**, **d**). Reprinted with permission from [89]. Copyright (2015) Springer Nature

﻿The X-ray diffractograms of all samples were almost identical, revealing no reflections as a result of the amorphous nature. Identical Ti 2*p3/2* and Ti 2*p1/2* peaks were also found in the XPS spectra, and no shifting, widening, or narrowing was observed, linked to the Ti^4+^ of the Ti–O bonds. Since no Ti^3+^ moieties exist in the matrix, all the obtained samples, regardless the color, were assumed as amorphous TiO_2_. The XPS analysis also showed the absence of other elements, rather than Ti and O, independent of the US irradiation and duration. The TEM and HRTEM images (Fig. 7) revealed that the obtained materials had an absolute disorder and amorphous structure, with or without US treatment. The same research team reported in a prior work the synthesis of hydroxylated amorphous and disordered TiO_2_ nanomaterials of different color shades [90]. The only difference was that instead of US irradiation, the obtained intermediate white powders were thermally treated in a muffle for 3 h (heating rate ~ 20 °C/min) at different temperatures; 200–800 °C. These nanomaterials, as also in the case of those reported by Chen et al. [87], had a specific structure: an anatase nano-core/shell surrounded by a disordered and amorphous hydroxylated phase. Contrarily, the US treatment led to core-free pure amorphous TiO_2_ nanocrystals. In order to exclude the possibility of the blackness being associated with N doping, NaOH was used as a base instead of ammonia, and the obtained materials showed similar blackness increment by the extension of US irradiation.

The initially white and all ultrasonictreated samples darker in color showed similarly shaped O1s XPS spectra. The peak was deconvoluted to two symmetric peaks, one assigned to Ti–O bonds (~ 530 eV) and the other to Ti–OH (530.9–532 eV). However,, the Ti–OH/Ti–O ratio of Gauss peaks was increased by increasing the US treatment duration. The amorphous white TiO_2_ had a Ti–OH/Ti–O ratio of 0.73, while for the black sample, the ratio was more than double (1.60). The authors determined the hydroxylation degree and assumed a molecular formula of TiO_2-*x*_(OH)_2*x*_, where “*x*” represents the hydroxylation extension. The reported molecular formulas were TiO_1.156_(OH)_0.844_ for the white powder and TiO_0.768_(OH)_1.232_ for the black sample. Calcination of all colored samples at 800 °C until a constant weight led to white powders, as a result of the transformation of Ti–OH to Ti–O. Based on all the above-mentioned results, it was concluded that the high-power US irradiation duration had a direct correlation to the hydroxylation and amorphism.

**Fig. 8:**
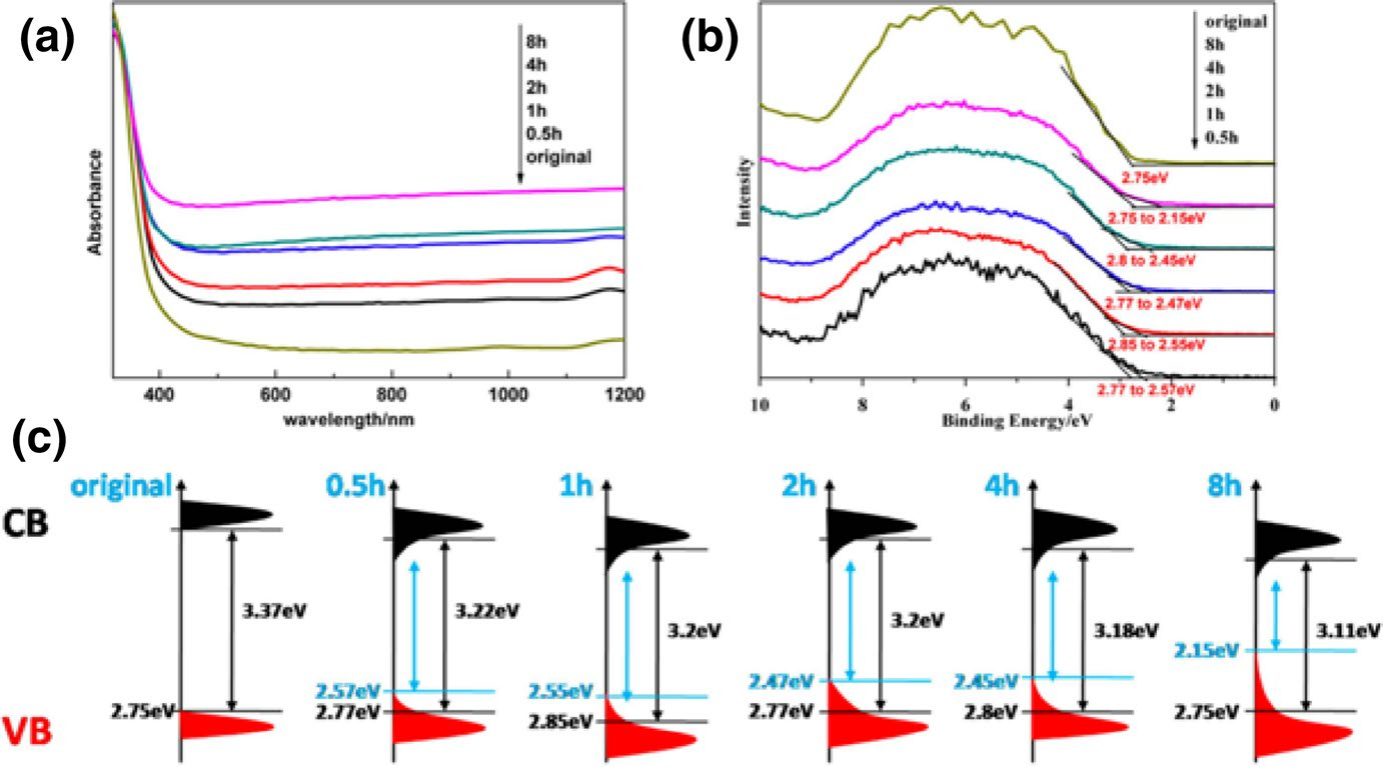
UV–Vis–IR absorbance spectroscopy (**a**), valance band XPS spectra (**b**), and a schematic illustration of density of states (DOS) of the original samples and amorphous hydroxylated samples ultrasonicated for different durations. The blue and black arrows indicate the bandgaps after and before localized band-bending, respectively. Reprinted with permission from [89]. Copyright (2015) Springer Nature

The increase of the hydroxylation and amorphism as a result of longer ultrasonic irradiation had additional positive impact on the desired, and ultimately key, features in catalysis due to improvement of light harvesting; the structural and optical features. The absorbance intensity through the whole visible and near-infrared regions was improved by the increase of the ultrasonication duration, while the bandgap was decreasing. The white and the black samples had a bandgap of 3.37 and 3.11 eV, respectively. The density of states (DOS) constructed by the optical absorbance and valance band XPS spectra (Fig. 8) showed that the narrowing of the bandgap was assigned to electronic structure alterations due to orbital overlapping, and the blue-shift of the valence band maximum towards the Fermi energy.

The increased porosity is also an important factor for photocatalytic application, since it enhances the reaction rates as a result of improved diffusion of the reactants and the availability of the active sites. For all studied samples, the obtained type IV nitrogen sorption isotherms with type H2 hysteresis loops revealed the existence of meso-pores/voids [91], resulted from the interstitial spaces between the nanoparticles, as was reported in various cases [80]. The lowest porosity values were found for the non-US-treated white powder; a surface area of 166.43 m^2^/g and total pore volume of 0.109 cm^3^/g. The US irradiation gave rise to the microporosity due to the hydroxylation of TiO_2_, as it was reported in other cases [92]. The black sample US-irradiated for 8 h showed the highest structural parameters, which were double compared to the white sample (surface area: 329 m^2^/g and total pore volume: 0.251 cm^3^/g).

The evaluation of the photocatalytic capability of the samples was performed by monitoring the decomposition/removal of acid fuchsin (AF) in aqueous solution. Since the materials were porous, the removal/reactivity in the dark was evaluated in detail prior the evaluation of photocatalytic performance. It was found that the black nanomaterial had an almost three times higher removal capability in the dark compared than the white one, due to the higher surface area and pore volume. The analysis of the interactions (by eliminating the effect of physical adsorption) showed that the US-assisted synthesis led to samples that possess an improved solar- and the visible-light-driven photocatalytic reactivity. The first-order rate constant obtained by the Langmuir–Hinshelwood model for the black sample was 5.8 and 7.2 times higher under solar and visible light irradiation, respectively, compared to the non-US-treated white sample. The decomposition capability was linked to the formation of hydroxyl radicals. The fact that the photocatalytic reactivity improvement was more pronounced in the case of visible light was linked to the enhanced light utilization/harvesting, photo-response range, and the narrowing of the bandgap. Photoluminescence tests showed that the increase of the ultrasonication duration led to a decrement of the photo-generated electrons and holes pairs, with the latter being trapped at the disordered phase.

### 1-D Particles

#### 1-D Titanium Oxide and Titania

**Fig. 9:**
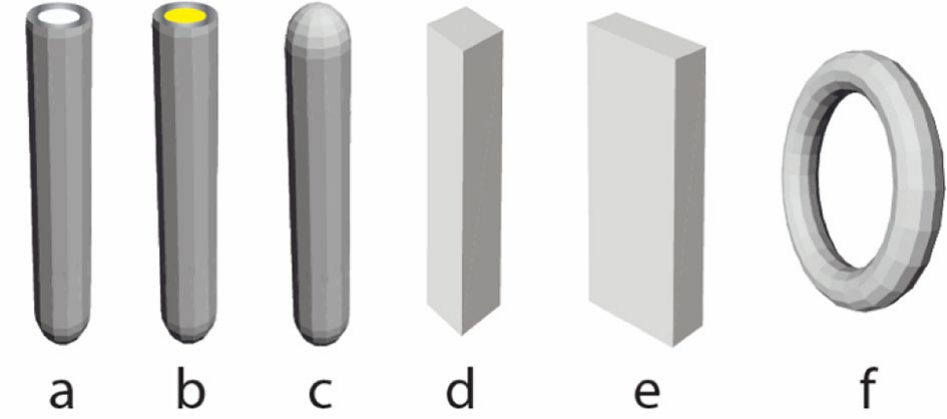
Schematic illustrations of the most widely synthesized and reported titanium oxide nanoscaled morphologies: open-end nanotube (**a**), core–shell nanotube (**b**), nanorod (**c**), square or rectangular nanorod/belt (**d**, **e**), and nanoring (**f**)

In the literature, various different names/terminologies are used for the characterization of the structure and shape of the 1-D synthesized materials, like fibers, whiskers, nanotubules, fibrils, nanocable, rods, nanowires, belts, since the definition and nomenclature are not well stablished [93]. The geometrical shapes of the titanium oxides that are more widely accepted, reported as a characteristic based on electron microscopy images, and herein used, are collected in Fig. 9. In general, the most important shapes are the open-end NTB (a), the core–shell NTB, the nanorod (c), the square or rectangular nanorod/belt (d, e), and the nanoring (f) [93].

**Fig. 10:**
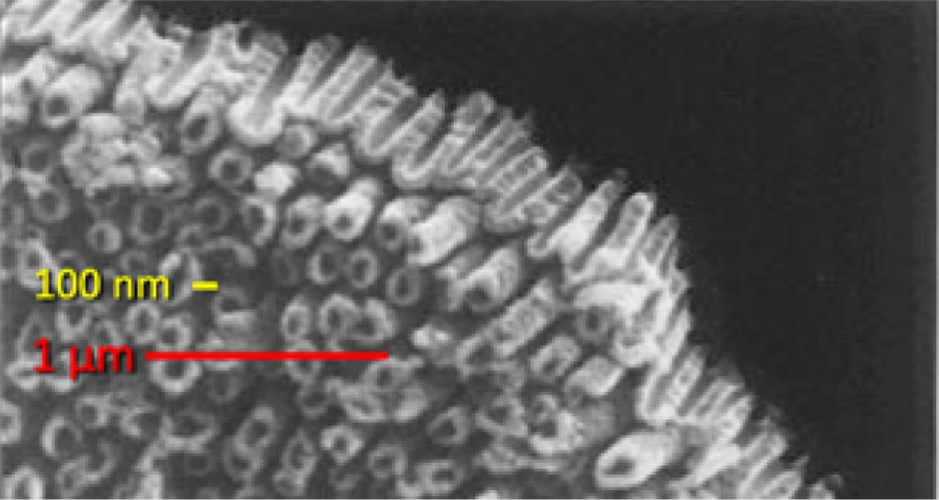
SEM pictures of the cross section of the as-prepared film of titania (with the upper part of the tubes removed). (Adapted from Fig. 3 of [81]). Reprinted with permission from [81]. Copyright (1996) American Chemical Society

The first report of 1-D TiO_2_ NTBs was by Patrick Hoyer in 1995 [81], who used a poly(methyl methacrylate) (PMMA) mold/template for the electrochemical deposition/growth of the titania NTBs. After the dissolution of the polymer, the obtain material consisted of poorly organized arrays of amorphous TiNTBs. The diameter of these NTBs was in the range of 140–180 nm, with an inner hole diameter of 30–50 nm and wall diameter of 30–50 nm. A 45° view of the cross section of the lower part of the amorphous tubes (after the removal of the upper part of the NTBs) is presented in Fig. 10. The electrochemical synthesis is out of the scope of this work. A detailed review article for the electrochemical formation of self-organized TiO_2_ NTBs was published by Roy et al. [94].

**Fig. 11:**
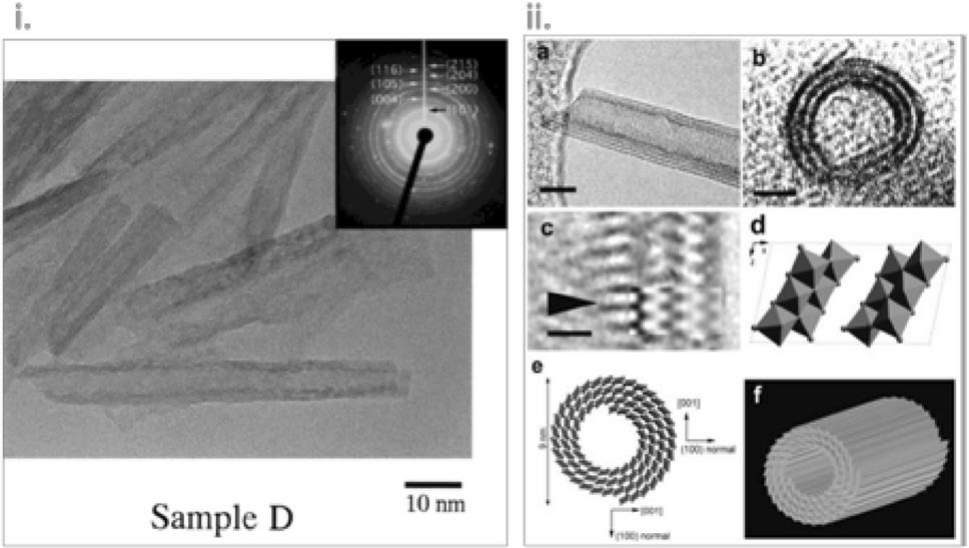
**i** ﻿TEM image and SAED pattern of titanate nanotubes hydrothermally synthesized the for first time by Kasuga et al.; **ii**: **a** HRTEM image showing a nanotube with an open end and three or four layers at the walls (scale bar 6 nm); **b** HRTEM image of the cross section of a three-layered wall nanotube (scale bar: 3 nm); **c** enlarged HRTEM image (scale bar: 1 nm); **d** a structure model of a single unit cell of H_2_Ti_3_O_7_ ([010] projection); **e** schematic illustration of the nanotube’s structure; **f** 3-D drawing of a titanate nanotube. Adapted with permission from [97, 101], respectively. Copyright (1999) and (2002) Wiley

The fascinating TiNTBs were bulkily and template-free firstly obtained in a powder form via the innovative work of Kasuga et al. in 1998 [95]. TiNTBs with a small diameter (Fig. 11i) were synthesized from the conversion of TiO_2_ (mixed rutile and anatase) by a soft chemical method; hydrothermal treatment (110 °C, 20 h) in a strongly basic environment (10 M NaOH). They showed by TEM how the treatment with diluted HCl can lead to nanotubular structures and of high specific surface area, up to 257 m^2^/g. Peng’s group analyzed in a series of articles in between 2001 and 2003 [96–99] the crystallographic structure of the hydrothermally obtained TiNTBs, and assigned it to trititanate H_2_Ti_3_O_7_. They also presented the catalytic role of NaOH and how the NTBs are formed by the rolling of the intermediately formed nanosheets (Fig. 11ii). In 2004, Suzuki and Yoshikawa expanded the analysis by proposing that the presence of water molecules is crucial [100], expressing the formula as H_2_Ti_3_O_7_.*n*H_2_O, and showed that these moieties of the crystallographic water play a role in the interlayer spacing of titanate layers of the NTB’s wall.

**Fig. 12:**
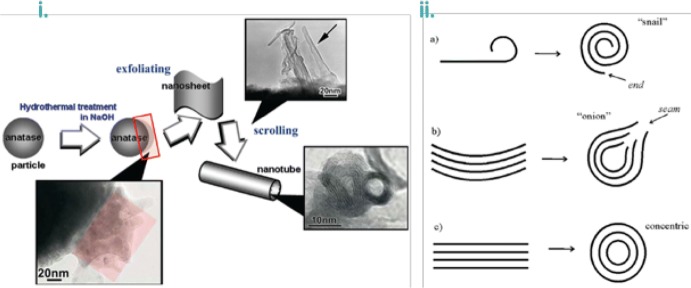
**i** A schematic collective scheme for the exfoliation and wrapping/scrolling of the formed titanate nano-sheets leading to the nanotubular particles, supported by TEM observations; **ii** a schematic representation showing the possible mechanisms for the formation of the nanotubes. Adapted with permission from [102, 103]. Copyright (2010) American Chemical Society and (2004) RSC, respectively

In general, this synthetic process involves two main steps, the first being a conventional solid-state reaction between TiO_2_ and sodium ions in basic solution, forming layered structures peeled from the initial particles. The second step involves the ion exchange during the acid treatment, HCl in almost all reported cases. Two factors are important regarding the formation of the alkali metal stabilized nanotubes: (1) how the nanosheets are formed from the spherical (in most cases) nanoparticles, and (2) how the nanosheets are converted to NTBs. Regarding the first aspect, Nakahira et al. showed by TEM observation (Fig. 12i) in 2010 that the formation by surface exfoliation of the nanosheets and their rolling/wrapping to NTBs take place on the surface, using as raw material an anatase-type titanium dioxide, and they proposed the entire process by various characterizations [102]. Bavykin et al. presented three different possible mechanisms of the conversion of the nanosheets to open-end multi-wall NTB, resulting in differently structured tubular shapes (Fig. 12ii) [103].

After the first reports of the TiNTBs, an intense research effort was focused on tuning different parameters during synthesis in order to control the structural and morphological features, the homogeneity and purity of the formed TiNTBs, as well as to decrease the synthesis temperature and duration [100, 102–110]. However, some arguments were derived. More details regarding titania NTBs obtained by hydrothermal-based synthesis can be found in the review article reported in 2011 by Wong et al. [111]. In many of the reports regarding the synthesis of the 1-D nanotubular structures, US irradiation was applied at different stages of the process, but without analyzing the possible role. It is feasible to believe that US led to specific effects that were not explored. Sonication can also help the characterization and separation of the TiNTBs. Interestingly, Bavykin et al. showed that US irradiation can be beneficial in order to distinguish the nature of the high observed pore volume by separating the agglomerates into individual NTBs [103].

The focus of the following part is on how the sonication can play a vital role for the manipulation of the TiNTBs’ important factors like size, shape, porosity, peeling of the nanosheets, and more, as well as how the process can be achieved faster with a more environmental and energy-friendly manner. The photocatalytic activities and the involved mechanisms, if reported, are also introduced and discussed.

#### 1D Titania by Ultrasound Irradiation

In 2001, Zhu et al. [112] demonstrated that the utilization of low-frequency US can promote the formation of 1-D titanate nanoparticles. The one-pot synthesis of the whiskers and nanorods was based on the sonication of synthesized titania nanoparticles in strongly basic solution (NaOH, 10 M), following by washing with dilute HNO_3_ (0.1 M) and deionized water and vacuum drying. Compared to other methods used for the synthesis of ﻿1-D structured titania ﻿(template synthesis, supra-molecular assembles, hydrothermal synthesis, and inductive synthesis), this synthetic approach taking place in a one-pot synthesis is faster, while avoiding the use and removal step of the templates and the need of calcination for crystallization as the last step.

The used synthesized TiO_2_ nanoparticles as precursors were prepared ﻿by hydrolysis of titanium butoxide, followed by calcination at 650 °C for 1 h. The average size of them was around 20 nm, ﻿while the crystallographic composition was 17% anatase and 83% rutile. For the synthesis of the whiskers, synthesized titanium oxide nanoparticles were dispersed in the basic aqueous solution inside a Teflon vessel. The mixture was ultrasonicated for 80 min (direct immersion of Ti-horn, 560 W_elec._, frequency not specified but probably in the low-frequency range, 20–80 kHz). The temperature during the synthesis was 80 °C.

**Fig. 13:**
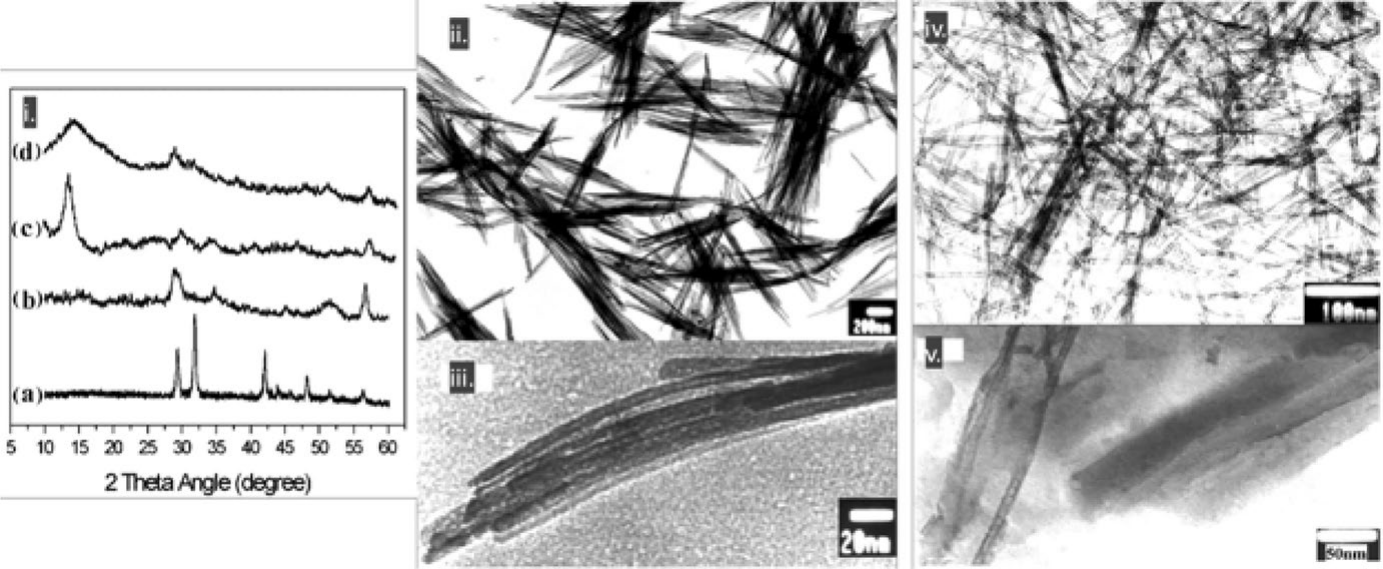
**i**: XRD patterns of titania particle precursors (a), titania whiskers (b), H_2_Ti_8_O_17_ whiskers (c), and nanotubes (d); TEM images of titanate (**ii**) and TiO_2_ whiskers (**iii**), titania nanotubes (**iv**), and sample obtained by thermal treatment (4 h, 110 °C) of the sonicated products followed by washing with water for 5 min (**v**). Adapted with permission from [115]. Copyright (2005) American Chemical Society

Then the mixture after sonication was washed with diluted HNO_3_ for 2 h and with deionized water for 6 h. The obtained particles had a slender sheet structure of a 60-nm diameter and a length around 1 μm. The interesting outcome arises from the elemental stoichiometry analysis, which was found to be ﻿H_3_Ti_3_O_7.5_. The bands at ~ 3400 and ~ 1630 cm^−1^ at the IR spectrum were linked to the stretching vibrations of the O–H bond and to bending vibration of H–O–H, revealing the presence of water. Since the XRD pattern matched with that of H_2_Ti_3_O_7_ [113], and taking into consideration the thermogravimetric results, the product was assigned from the authors as H_2_Ti_3_O_7_.0.5H_2_O. Further washing of the product with water for 8 h led to nano-whisker arrays of a 5-nm diameter. The X-ray diffractogram revealed that the crystallographic phase changed to TiO_2_ (B) [114] (Fig. 13).

For the preparation of the NTBs, the mixture was treated with half the US power (280 W_elec._) for 60 min, and afterward, the Teflon vessel was maintained in an oil bath at 110 °C for 4 h. The washing was with HNO_3_ (0.1 M, 2 h) and deionized water (14 h). The obtained NTBs had a 5-nm diameter and 200–300-nm length. The XRD analysis revealed that the ﻿crystallographic phase was an intermediate between H_2_Ti_3_O_7_·0.5H_2_O and TiO_2_ (B). No Na was detected at the elemental analysis, while the ratio of Ti to O was 1:2.

The proposed mechanism of the whisker formation was based initially on the US-assisted reaction of the base that leads to the cleavage of some Ti–O–Ti bonds. The formed layered titanate lattices have octahedral form with alkali metal ions to occupy the interlayered regions. During the washing with acid and water, ion exchange and dehydration occur, resulting to H_2_Ti_3_O_7_·0.5H_2_O. Extended dehydration by water washing promotes the transformation to titanate bronze. The role of US is vital since it promotes the reaction between the raw nanoparticles and the base, as well as controls the oriented growth. The synthesis is faster by the application of US compared to the reported hydrothermal methods of nanorod formation. It is worth mentioning that without ultrasonication, no whiskers were obtained. A lower US irradiation power and the hydrothermal treatment promotes the formation of bigger titanate sheets and the exfoliation of nanosheets. The latter roll into NTBs during the washing due to the removal of the ions and, as a result, to alterations of the electrostatic forces/equilibria. Even though the above US-assisted hydrothermal approach successfully led to the preparation of NTBs significantly faster and easily compared to the hydrothermal synthesis, there is a drawback. The synthesis of the precursor starting with titanium butoxide hydrolysis is time-consuming and complex.

**Fig. 14:**
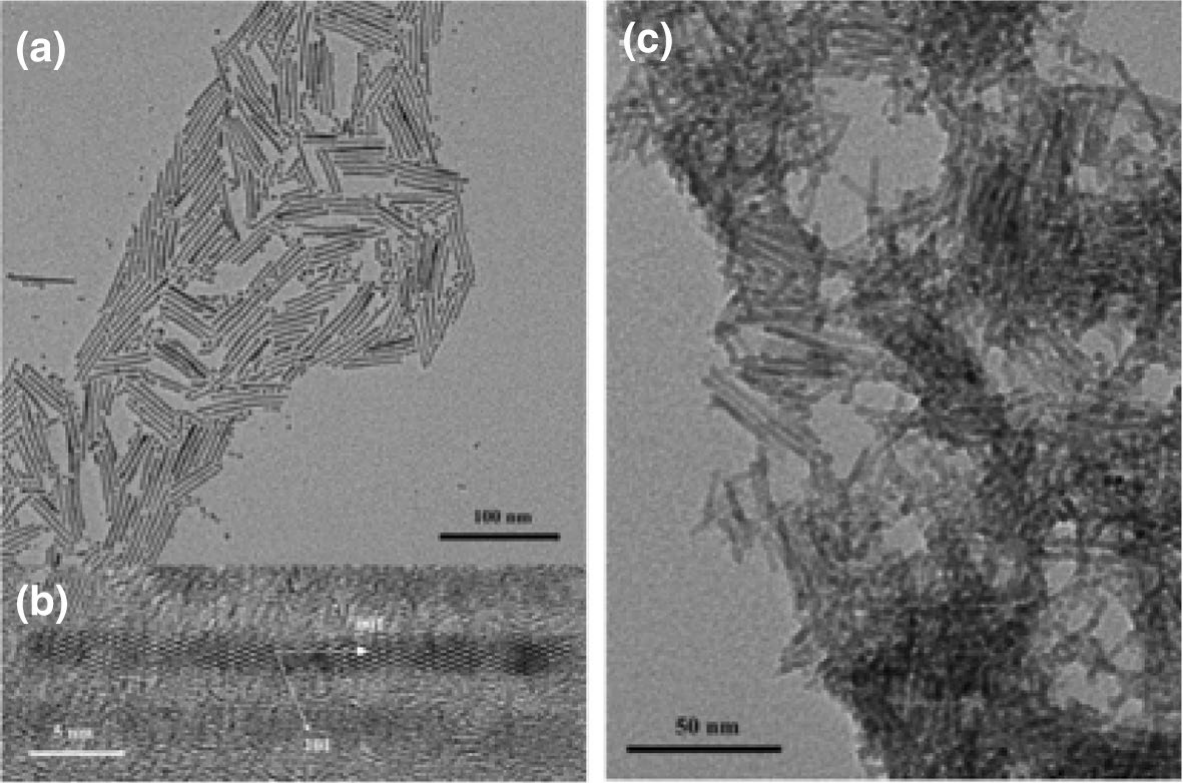
TEM and HRTEM images of the as-synthesized TiO_2_ nanocrystals prior the size-selective separation (**a**, **b**) and TEM image of the final TiO_2_ nanorods (**c**). Reprinted with permission from [115]. Copyright (2005) American Chemical Society

In 2005, Joo et al. [115] reported the synthesis of TiO_2_ nanorods of a diameter and length of 3.4 and 38 nm, respectively, by a nonhydrolytic ester elimination reaction between titanium(IV) isopropoxide (TTIP) and oleic acid [OA, CH_3_(CH_2_)_7_CH=CH(CH_2_)_7_COOH]. The latter monosaturated fatty acid is among the most common fatty acids in nature, produced both from vegetables and animals, and in this work, it was utilized as surfactant and shape stabilizer during the synthesis. Even though the authors concluded that the obtained crystallographic phase was of anatase, they did not report the region of the XRD for angles lower than 20°. In the following preparation method, TTIP was added to OA, and the suspension was heated gradually until 270 °C within 20 min and was kept at this temperature for 2 h. The initial clear solution of a yellow shade turned progressively to white. The yield was around 70% wt, and the white powder consisted of nanorods and ﻿quasi-spherical nanoparticles ~ 3-nm diameter (Fig. 14a, b). Interestingly, the authors were able to separate the nanorods by conducting a size-selective precipitation from a hexane/ethanol solution (Fig. 14c). They also showed that the nanorods’ diameter could be controlled by adding different amounts of 1-hexadexylamine. Sonication for 30 min (experimental conditions not specified) was applied for the removal of the surfactant after the treatment of the powder with superhydride solution (lithium triethylborohydride in THF), but the effect of US was not explored. ﻿The finally obtained nanorods presented a specific surface area of 198 m^2^/g and they were highly dispersible in water, a fact of a paramount importance for real-life applications. The estimated bandgap of the nanorods was 3.33 eV, a value higher than that of 3.2 eV of the bulk anatase, due to ﻿quantum size effect. Compared to commercial TiO_2_ P25, the obtained nanorods were found to possess a higher photocatalytic inactivation capability against *E. coli*, a fact that was assigned by the authors to the increased bandgap, surface area, and amount of surface hydroxyl groups. The improved and faster inactivation performance for the nanorods was linked to the elevated hydroxyl radical formation.

﻿ Since the morphological and structural features of TiNTBs (size and porosity) can be controlled, the application of US irradiation towards the increment of these features gained more attention. Ma et al. [116] reported in 2006 the synthesis of longer NTBs with a smaller diameter by a combined sonication-hydrothermal approach and using as precursor the commercial TiO_2_ P25. Their approach was based on dispersing the commercial powder in a Teflon vessel filled with NaOH aqueous solution (10 M). Using an immersed titanium horn (probably low-frequency, not specified), the suspension was sonicated at 70 °C under different sonication powers (100, 280, and 380 W_elec._) and varying also the duration (15, 30, and 60 min). The vessel was placed in a stainless-steel autoclave for hydrothermal treatment for 4 h at 110 °C. The obtained precipitate was washed with HCl (0.1 M) and deionized water until an acidic pH, centrifuged, and dried under vacuum. The role of the precursor on the size of the NTBs was determined by using different commercial TiO_2_ precursors.

**Fig. 15:**
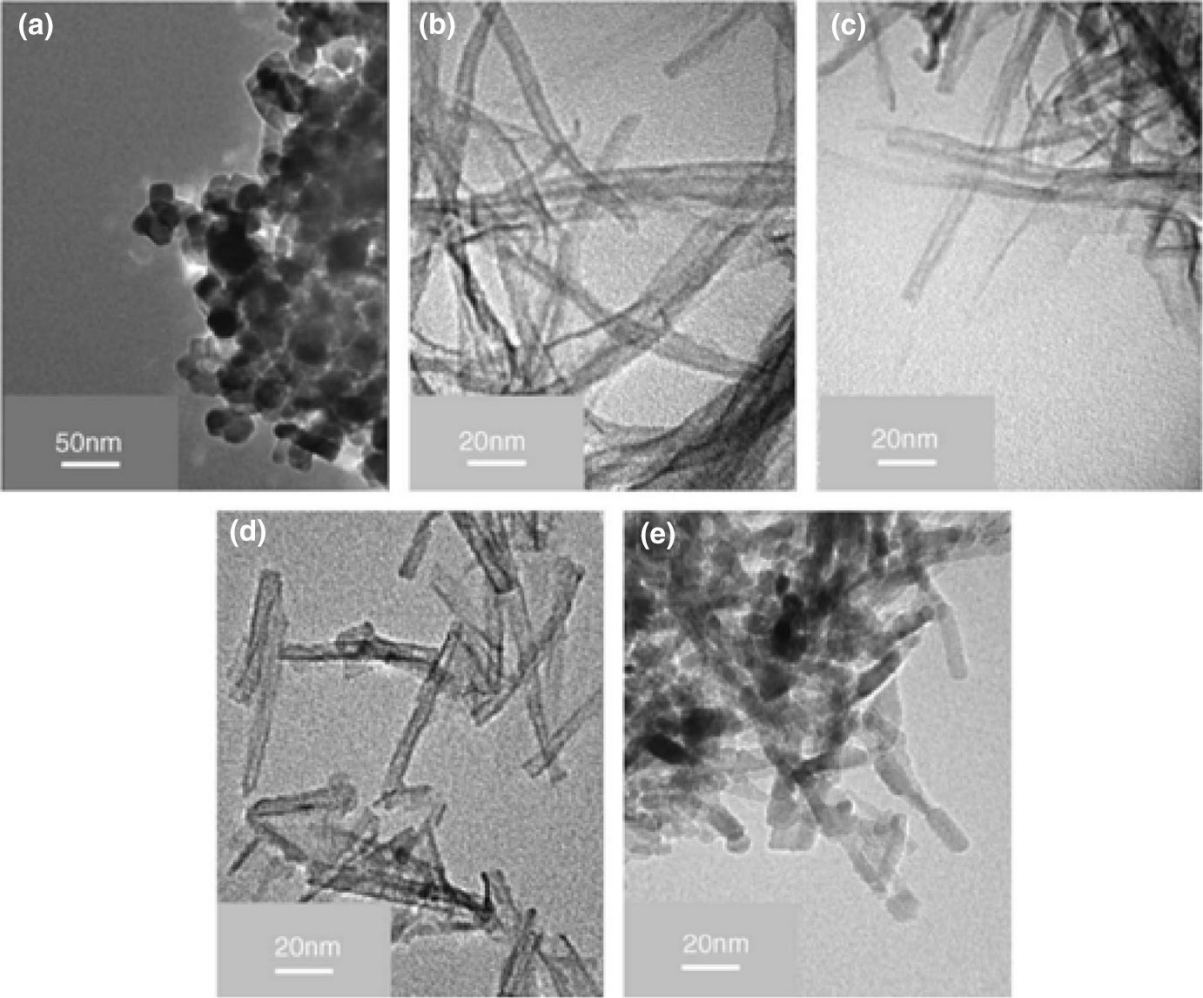
TEM images of TiO_2_ P25 precursors (**a**), titanate nanotubes as received (**b**), after calcination at 300 °C (**c**), at 450 °C (**d**), and 600 °C (**e**). Adapted with permission from [116]. Copyright (2006) Elsevier

The hydrothermal treatment of the nanospherically shaped P25 particles of an average size of ~ 30 nm without ultrasonicated pre-treatment led to minimal particle shape alteration. Sonication for 1 h prior the hydrothermal treatment with powers of 100 and 280 W_elec._ resulted in sheet and fibrous morphologies, respectively. A typical tubular morphology was achieved (diameter: 9–14 nm and length: 100–600 nm) by sonication at a higher power (380 W_elec._), revealing that the sonication, as well its power, plays a key role in the desired transformation to TiNTBs. TEM images of the initial TiO_2_ P25 and the TiNTBs produced by combining high-energy sonication and hydrothermal treatment can be seen in Fig. 15.

The crystallinity and the chemical composition of the nanorods were investigated by XRD and energy-dispersive spectroscopy (EDS) analysis. It was concluded that the chemical composition was ﻿H_2_Ti_4_O_9_.H_2_O (JCPDS 36-0655), while traces of rutile fractions were also observed. Considering that the TiO_2_ P25 consisted of 70% anatase phase and around 30% rutile phase, the transformation of the latter phase is preferable, while the former type is more stable under the hydrothermal treatment conditions. The EDS elemental analysis showed the absolute absence of Na.

The authors also studied the effect of the higher sonication power (380 W) without the hydrothermal and acid treatment. 15 min of US irradiation did not reveal the ability to alter the shape of the spherical particles. Increase of the irradiation time to 30 min led to swelled nanoparticles with an average diameter of 100 nm, probably as a result of the spherical particles merging. By increasing the duration of irradiation to 60 min, the observed morphology was found to be nanorods like, with lengths in between 100 and 300 nm. By hydrothermal and acidic treatment after the 60 min of US irradiation, the length of the nanorod-like particles increased up to 600 nm. Additionally, the diameter of the tubes was also smaller, but the shape homogeneity was not so perfect. Based on these observations, it can be proposed that the US effects can originate the reaction of the TiO_2_ nanoparticles with the base, by promoting the cleavage of the Ti–O–Ti lattice bond and the intercalation of Na^+^ at the lattice. The spherical ﻿forms are swollen and transformed to nanorods by increasing their length. Calcination at 300 and 450 °C of the sample obtained after the two-step process was not accompanied with notable shape alterations (Fig. 15c, d). On the contrary, calcination at 600 °C led to morphology transformation of the hollow tubular structures to rod-like structured nanoparticles (Fig. 15e).

**Fig. 16:**
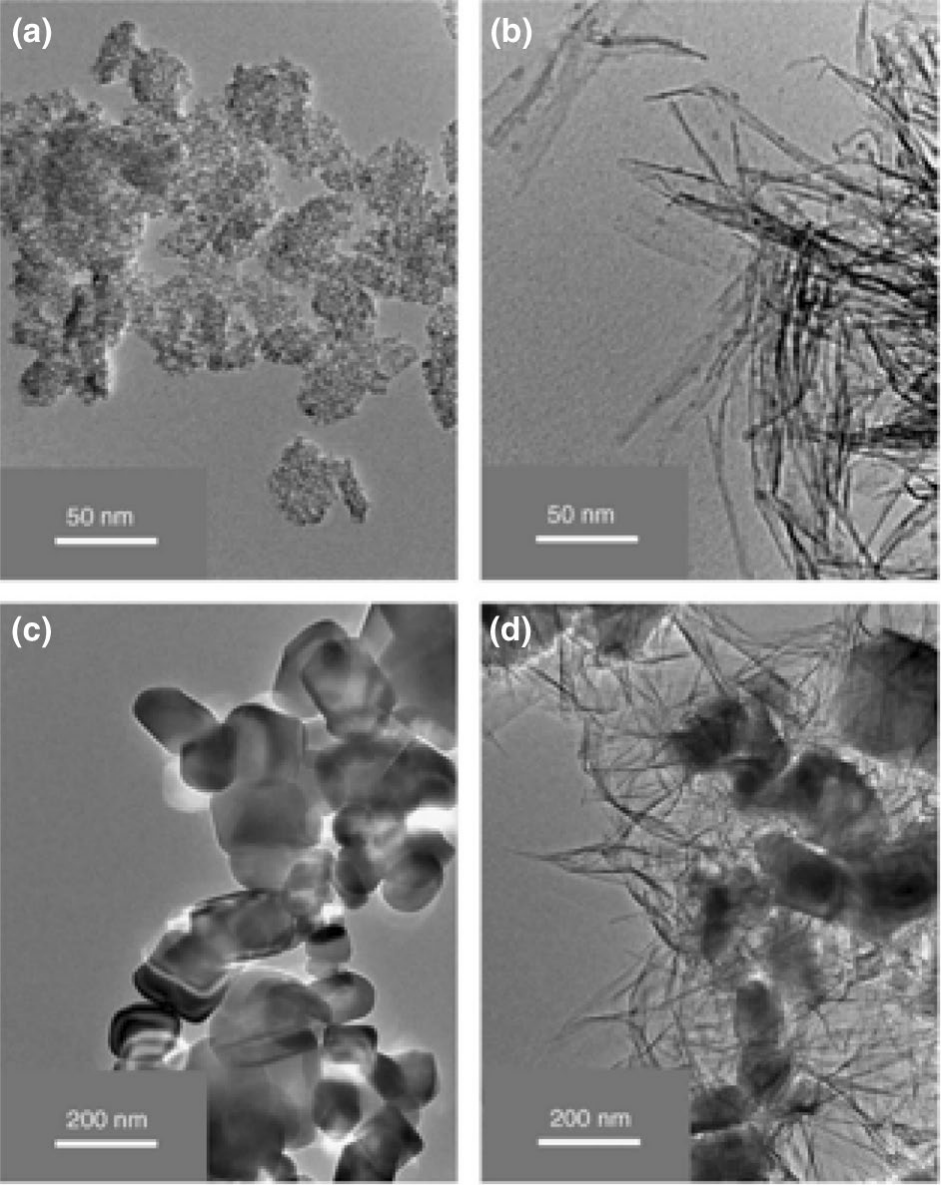
TEM images of TiO_2_ Hombikat UV100 precursor (**a**), titanate nanotubes derived by sonication-hydrothermal treatment of Hombikat UV100 (**b**), TiO_2_ BCC100 precursor (**c**), and sample obtained from BCC100 (**d**). Adapted with permission from [116]. Copyright (2006) Elsevier

Interesting outcomes regarding the vital role of the precursor's particle size were derived by using two other commercial TiO_2_ powders instead of P25. When the size of the initial particles was around 10 nm (Hombikat UV100, Fig. 16a), the formed NTBs had inner and outer diameters and lengths of 3–6, 7–10, and up to 400 nm, respectively (Fig. 16b). When particles of a bigger average size of 200 nm (BCC100, Fig. 16c) were used as precursor, instead of tubular-shaped particles, sheet-like structures with rolled edges were obtained together with untransformed particles that were slightly changed in size and shape (Fig. 16d). This was linked to the fact that the formed sheet-like structures cannot transform/roll to tubes, perhaps due to a hindrance effect by the larger particles.

Tanthapanichakoon and his colleagues showed and analyzed how the ultrasonication pretreatment can influence controllably the length of the titania NTBs [117, 118]. Interestingly, they used a commercial precursor (KISHIDA) of a low specific surface area (8 m^2^/g) and relatively large particles (400 nm) compared to the previous reports. By using a titanium horn (probably low-frequency, not specified in the article), the suspension of TiO_2_ in a 10 M NaOH aqueous solution was sonicated prior the hydrothermal treatment for 8 min with different supplied powers, from 0 to 38.1 W. After thermal treatment for 3 days at 150 °C, the obtained suspension was treated/washed with HCl and H_2_O. TEM analysis revealed that no US irradiation led to TiNTBs (herein referred to as short) with multilayered walls (2–6 layers and ~ 0.8-nm interlayer spacing), diameters from 4–6 nm, and lengths between 30 and 200 nm. Sonication by two different powers, of 7.6 and 38.1 W, led to increase of the TiNTBs' diameter. The majority of the NTBs had a length above 300 nm, while the diameter was in the same range with that of the short ones. Based on the previous analysis by XRD of the *d*-spacing between the adjacent layers of the tube walls by Suzuki and Yoshikawa, the authors concluded that the US irradiation and the resulted increase of the length was not accompanied with an interlayer spacing change. Additionally, the strong intensity diffractions of the initial TiO_2_ (anatase phase) were totally diminish in all synthesized TiNTBs.

﻿The dynamic light scattering (DLS) results showed average sizes of 53, 490, and 1760 nm for the samples prepared under 0, 7.6, and 38.1 W, respectively. Additionally, the size distribution was very narrow in the case without US irradiation, and the distribution was dramatically increased by increment of the applied US power. The formation of NTBs in size many folds higher the pristine particles can be linked to bigger peeled nanosheets prior the rolling, and/or to the connection of the formed tubes. An increasing trend was found between the specific surface area (*S*_BET_) and the power of US irradiation. The raw powder had an *S*_BET_ of 8 m^2^/g, and the short NTBs 179 m^2^/g. The US wave exposure at the pretreatment stage led to *S*_BET_ of 258 and 245 m^2^/g, for US power of 7.6 and 38.1 W, respectively. The positive effect of ultrasonication is via the enhancement of the de-aggregation of the particles, resulting in the peeling thorough swelling of large nanosheets that role to NTBs [118]. Without US waves, the size of the peeled nanosheets and, as a result, the size of the formed NTBs is smaller.

The same research team studied (in 2009) the effect of different preparation variables and combinations like particles size of the raw TiO_2_ (400 nm and 1 μm), temperature during the synthesis (90–180 °C), and sonication power, with valuable conclusions on how these variables can adjust the morphological and structural features [117]. The hydrothermal treatment at 150 °C without sonication of the commercial TiO_2_ particles of size ~ 400 nm led to NTBs of an average length of 79 nm and a specific surface of 179 m^2^/g. The respective values were 143 nm and 118 m^2^/g when the largest (1 μm) raw particles were used. This was linked to the formation of bigger but less in number intermediate sheets during the peeling. By studying the effect of different temperatures (90, 120, 150, and 180 °C), it was concluded that the transformation of titanium dioxide to titanate was complete even at 120 °C. However, increase of the temperature to 180 °C led to a shift of the characteristic diffraction at around 10° 2*θ* to a higher angler (~ 12°), suggesting a narrow interlayer spacing between the layers of walls. Suzuki and Yoshikawa assigned the characteristic XRD reflection peak at 2*θ* = ~ 10° of the hydrothermally synthesized H_2_Ti_3_O_7_.nH_2_O NTBs to an interlayer distance of 0.92 nm [100]. An interesting parenthetical fact can be added at this point. The high-temperature XRD pattern obtained at 100 °C was almost identical with the one at room temperature, but at 200 °C, the reflection was shifted to 11.2°. This narrowing of the interlayer space to 0.79 nm was linked to the removal of the water moieties between the layers of the wall. It is worth mentioning that thermogravimetric analysis of the NTBs showed that above 200 °C, the weight loss was very limited.

**Fig. 17:**
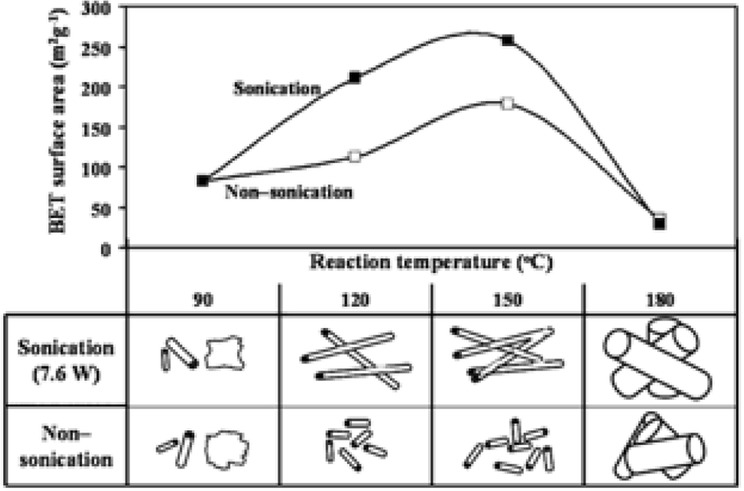
BET specific surface area and morphology/shape of titanate products synthesized (from raw TiO_2_ of an avg. size of 400 nm) at reaction temperature of **a** 90 °C, **b** 120 °C, **c** 150 °C, and **d** 180 °C without or with US irradiation (power of 7.6 W). Reprinted with permission from [117]. Copyright (2009) Elsevier

Going a step further, the team of Tanthapanichakoon [117] chose to study the effect of temperature during the synthesis with or without US pre-treatment by using raw particles of an ~ 400-nm diameter, due to the fact that this raw TiO_2_ gave higher *S*_BET_ compared to the raw one with average particle size of 1 μm. The resulted specific surface areas and the morphology of the sample are presented in Fig. 17.

**Fig. 18:**
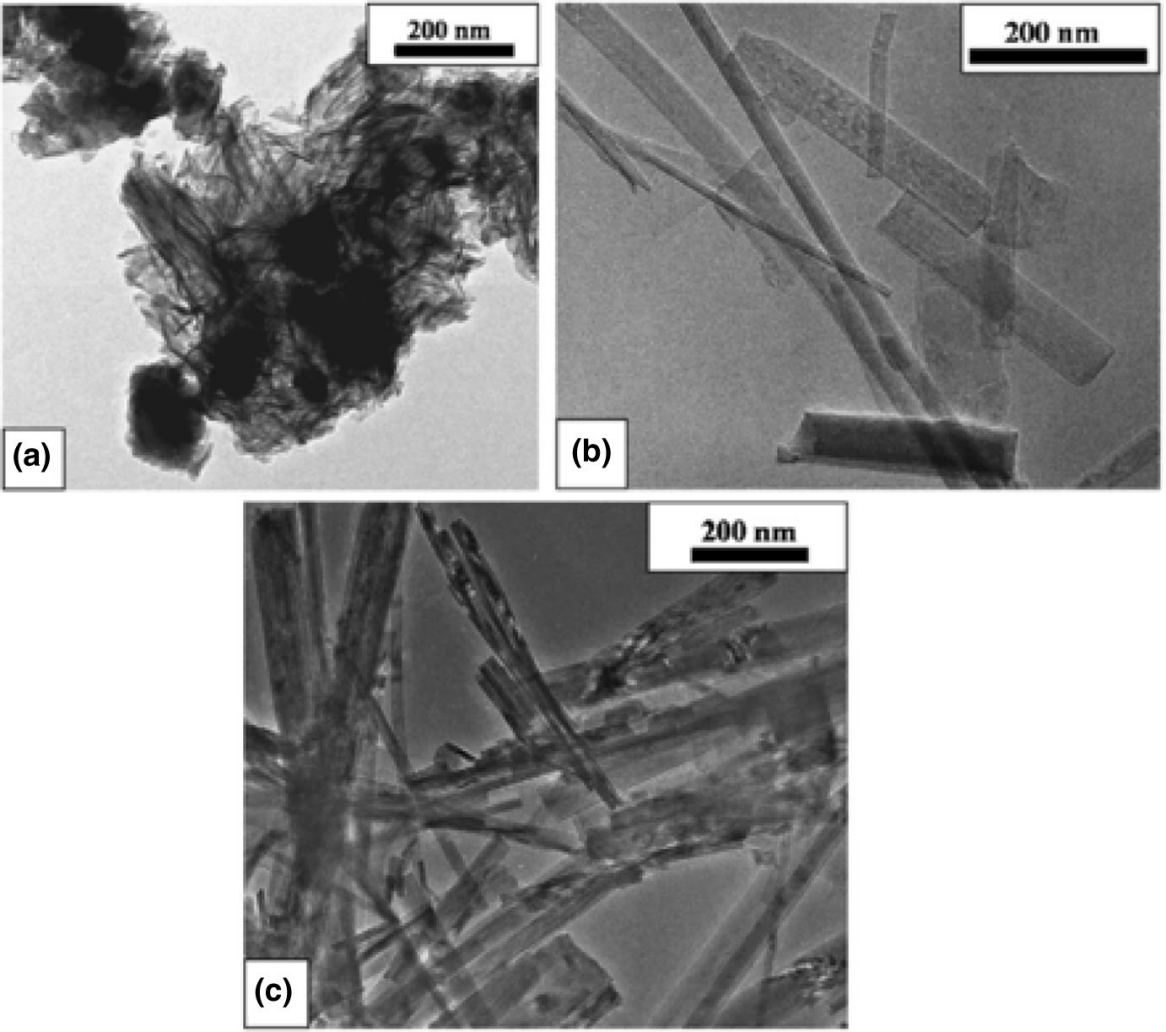
HRTEM captures of mixed titanate nanostructures obtained from raw TiO_2_ (of an average particle size 400 nm) at reaction temperature of 90 °C (**a**) and titanate nanofibers/wires synthesized at 180 °C without sonication (**b**), and with power of 7.6 W (**c**). Adapted with permission from [117]. Copyright (2009) Elsevier

As can be observed, the effect of US pretreatment on the structural and morphological features is loud and clear, and, additionally, it had a key effect on the product purity and shape homogeneity, as confirmed by microscopy analysis. At 90 °C, NTBs, nanosheets, and remaining un-transformed crystals were detected either without or with US pretreatment (Fig. 18). Moreover, the use of sonication did not lead to higher *S*_BET_. The effect of US was dramatically more pronounced at a synthesis temperature of 120 °C. The length of the NTBs was much higher and the *S*_BET_ almost doubled in value. The purity was also enhanced, since no un-transformed crystals were detected after US pre-treatment. Analogous outcomes were derived when the synthesis was performed at 150 °C after US pre-treatment. Further increase to 180 °C had a negative impact on the *S*_BET_ and the desired morphology, with the US irradiation not leading to a specific effect. The predominant shape of the particles was of nanowires/fibers/rods in both cases, although with a smaller diameter in the case of US irradiation. The shape change was in good agreement with the angle shift of the XRD pattern, as was discussed above. It can be suggested that the thermal effects when the synthesis temperature is higher than 150 °C overcome the effects of the US pre-treatment.

## PART B

### Ball-Milling-Derived Nanomaterials

The utilization of ball milling (BM) in order to obtain TiO_2_ nanoparticles includes different possible pathways with regard to the used raw material. The latter can be either elemental Ti, either TiO_2,_ or a different source of titanium like a mineral. The duration and the power of the BM plays a crucial role, as does the atmosphere in which the process takes place. The achievement of high temperature is found to be in some cases a drawback, and for this reason, the ball milling is performed with breaks or even with the use of a liquid phase. The latter case is referred to as wet ball milling. In most reported cases and especially towards the formation of one dimensional TiO_2_ nanostructures, ball milling was utilized as a mechanochemical pretreatment to obtain metastable polymorphs, that can be further tuned in morphology by annealing or wet chemistry. In this part, we collected some reports in which the application of ball milling dramatically affected the final properties of the nanomaterials. Starting with 0-D nanomaterials and ending with 1-D materials, we tried to introduce the reported results following a chronological order. An emphasis was given when the nanomaterials were found to possess an elevated photocatalytic capability.

### 0-D Ball-Milling-Derived Nanostructures

In 1994, Begin-Colin et al. studied the polymorphic transformation of TiO_2_ from an anatase phase to a rutile phase by ball-milling (BM), based on XRD and Fourier transform infrared (FTIR ) spectroscopy techniques [119]. They observed that the phase transformation was not direct, since different transient phases appeared, with the one of type II being predominant. However, no electron microscopy analysis was performed. The importance of this study was the conclusion that the anatase-to-rutile transformation is not a direct process. Based on that, the authors emphasized that the ball-milling technique is feasible to obtain alloys with various non-equilibrium crystallographic phase materials. The intermediate crystallographic phases can further be tuned with various methods, in order to obtain desired photocatalytic properties.

An ultimately serious drawback of the ball-milling technique, especially when the target is a material of a high purity, is the possibility of atmospheric nitrogen incorporation into the structure or metal (predominately iron) from the used ball-milling apparatus. For instance, it was showed by Lu et al. [120] that, depending the atmosphere and the duration of the ball milling, different doping of N or Fe could result, even under air. More interesting, titanium oxynitrile instead of oxide can be obtained within a closed ball-milling system and extension of the mechanochemical process for up to 90 h. In 2007, Pang et al. [121] showed the possibility to synthesize a composite of ﻿titanium and hydroxyapatite by a wet ball-milling method. Hydroxyapatite (HP), Ca_5_(PO_4_)_3_(OH), is a natural mineral, while modified forms of HP are the main compounds of human bones and teeth. The increase of milling duration led to the decrease of the grain size, as well as to ﻿an improvement on the homogenously distribution of nano-hydroxyapatite. Analogous composites were synthesized by ball milling the same year by Silva et al. starting with ﻿Ca(H_2_PO_4_)_2_ and TiO_2_ as the raw materials [122].

In 2000, Begin-Colin et al. studied in details the kinetics and mechanisms of phase transformations induced by ball milling in air, starting with a commercial anatase TiO_2_ [123]. They concluded that the anatase is transformed by BM to rutile via a TiO_2_ II phase. The powder-to-balls ratio of weights (R) influenced the transformation rate. Regarding the nature of ball-milling media, the transformation’s kinetics were found faster in the case of alumina compared to steel.

﻿Yadav et al. showed in 2015 the synthesis of titanium oxide nanoparticles from elemental powder of Ti (~ 0.5 mm) by ball milling for 10 h [124]. The size of the spherically shaped particles was between 10 and 20 nm, while XRD analysis indicated a pure rutile phase. The estimated bandgap was 4.46 eV. They used the obtained material in order to form a solid-state sensor by pelletization and application to an Ag–pellet–Ag electrode configuration. This sensor was found to possess a sensitivity toward liquefied petroleum gas (LPG). The author linked this to the blue-shift of the optical bandgap and to the nanoscaled morphology of the obtained ball-milling-derived nanoparticles.

In 2016, Rejender and Giri [125] presented an anomalous strain-evolution, crystallographic phase alteration, and bandgap narrowing by strain engineering using ball milling ﻿and commercially available TiO_2_ powder as the precursor (particle size around 80 nm and bandgap 3.14 eV). Except for the decrement in size to 7–18 nm, the finally obtained TiO_2_ nanocrystals (NCs) found to obtain a new crystallographic phase of Ti_3_O_5_, as well as a narrow bandgap of 2.71 eV.

**Fig. 19:**
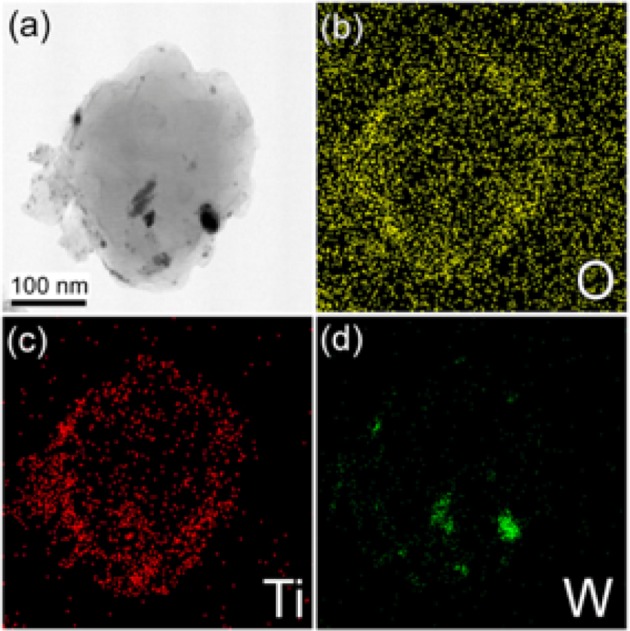
TEM image (**a**) and EDX maps (**b**–**d**) of TiO_2_-coated boron particles wet-milled for 8 h. Reprinted with permission from [126]. Copyright (2016) MDPI

Another interesting application of the wet ball-milling process was reported the same year by Jung et al. [126] for the TiO_2_ nano-coating of boron particles. Briefly, a tungsten carbide milling jar was filled with titanium(IV) isopropoxide, boron powder (average particles’ size ~ 800 nm), and hexane inside a glove box filled with nitrogen. The as-received suspension was further treated and washed with ethanol inside an US bath. They found that increase of the milling duration can lead to decrease of the final particle size, even up to ~ 150 nm. The particles were coated with an amorphous titania-containing layer (estimated 10 nm). The drawback of the extension of the ball milling was the incorporation of impurities, predominately tungsten, from the jar and balls, as can be seen in energy-dispersive X-ray (EDX) analysis (Fig. 19). The ball-milling-derived TiO_2_-coated nanoparticles were promising for hydrogen and oxygen evolution reactions (HERs, OERs) in photoelectrochemical applications.

﻿Another strategy to apply ball milling during the formation of TiO_2_ nanoparticles was by mixing and ball milling of different precursors [36]. In 2007, Billik et al. used either TiCl_4_ with (NH_4_)_2_CO_3_ or TiOSO_4_·*x*H_2_O with Na_2_CO_3_ [127, 128]. After ball milling, they received amorphous samples, and they linked this to no crystallization having occurred. After annealing, they obtained well-crystalized materials, with higher photoreactivity compared to P25, determined by electron-paramagnetic-resonance (EPR) studies. They reported also that the presence of Fe impurities plays a role in the photoactivity of the final material. In 2008, Salari et al. also used TiOSO_4_·*x*H_2_O as the Ti source but NaCl as diluent [129].

### 1-D Ball-Milling-Derived Nanostructures

An important, abundant, and cheap source used for the industrial production of bulk TiO_2_ is the iron–titanium oxide mineral (FeTiO_3_) mineral, known as ilmenite. A high amount of ilmenite exists in the Earth’s crust on all five continents, and on the Moon. The price of the raw material was around 80–107 USD per metric ton in 2004, while a peak was achieved in 2012 reaching even 350 USD per ton. In recent years, the cost has been around 250 USD/ton. The global demand has grown moderately in recent years, since it was estimated at around 6.4 million tons in 2010 with a prediction to reach above 8 million tons in 2025. The industrialized production of bulk TiO_2_ from minerals is based on chloride or sulfate processes. In recent decades, there has been increased research effort to expand the use of this mineral in order to prepare nanostructures of TiO_2_. The utilization of ball milling in order to promote the formation of nanostructured TiO_2_ for a “real-life” application by using ball milling dates from 2008.

﻿Li et al. (2008) [130] reported the formation of meso- and/or micro-porous hydrolysate TiO_2_ by an initial mechanical activation of ilmenite using BM, following by a simultaneous dissolution and hydrolysis in a dilute sulfuric acid aqueous solution. The effect of the acid concentration played a key role in the structural parameters, with 10% sulfuric acid leading to a surface area of 258 m^2^/g. In order to obtain the rutile-phased final material, calcination was applied. The importance of this work was that the ball-milling pretreatment made feasible the dissolution of the mineral in a dilute acidic solution. For an efficient decomposition in pigment production without mechanochemical utilization, an H_2_SO_4_ solution of a concentration above 80 wt% is required [130].

﻿In 2011, Tao et al. prepared flower-like FeTiO_3_ by pretreatment of ilmenite with high-energy BM followed by mild hydrothermal treatment in basic aqueous solution (1 M NaOH) [131]. They stated that the nano-petals comprising the final obtained flower-shaped particles had a thickness of 5–20 nm and sizes 100–200 nm. The hydrothermal treatment at 120 °C, even with 2 M NaOH, did not lead to noticeable changes in morphology. The obtained materials showed attractive capacitance values. Considering the above observations regarding the formation of NTBs, we can derive two possible conclusions/proposals. First, the presence of Fe stabilizes the layered structure of the nano-petals to roll to tubes. Second, the utilization of BM promotes the peeling of the mineral’s particle even at a lower concentration of 10 M, necessary for the hydrothermal peeling of TiO_2_ particles.

**Fig. 20:**
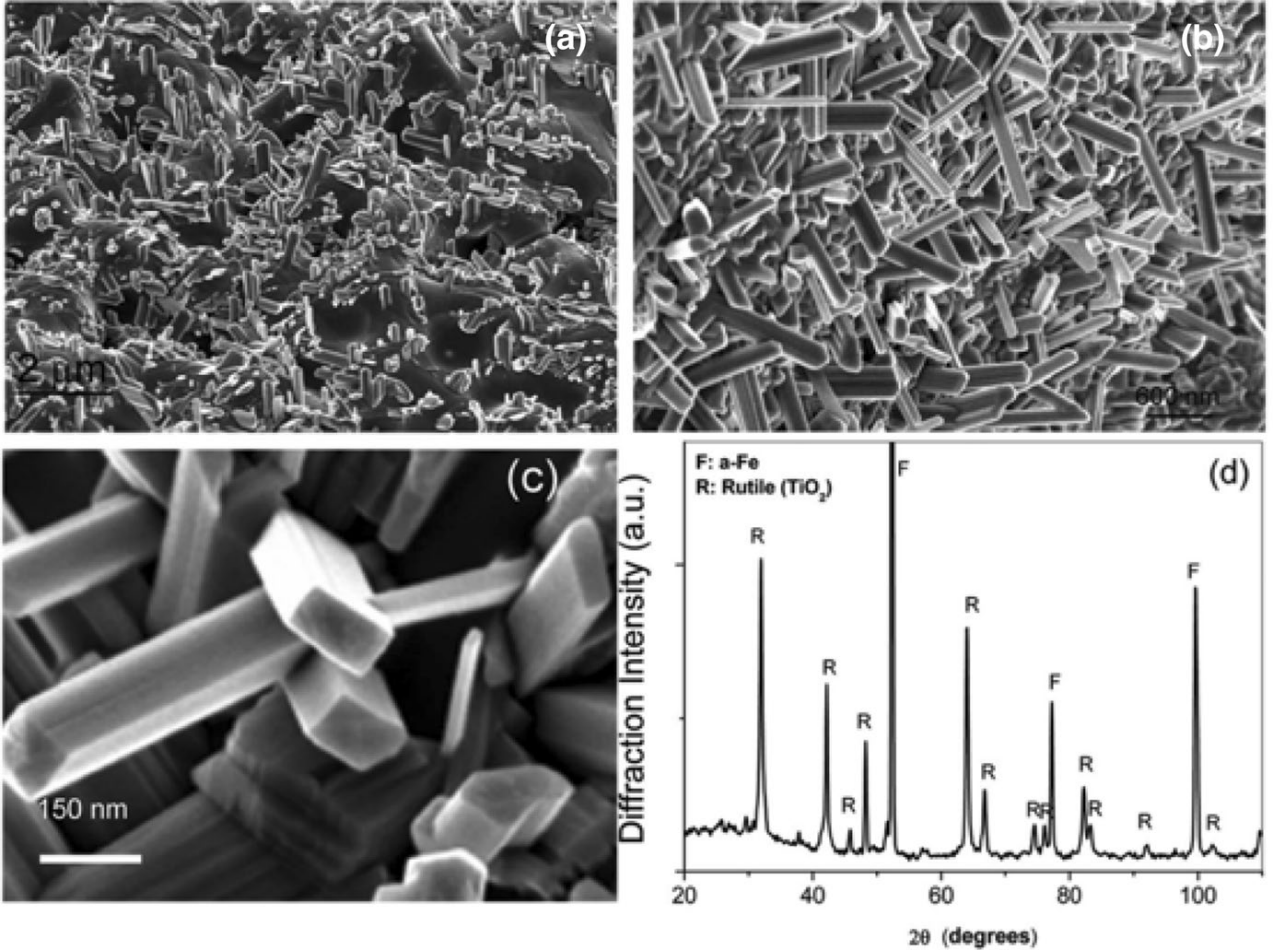
SEM image after the second annealing step at 700 °C for only 4 h (**a**), SEM image (**b**), a cross section of nanorods (**c**), and the XRD spectra (**d**) after annealing for 8 h at 700 °C. Adapted with permission from [133]. Copyright (2009) American Chemical Society

The formation of the 1-D nanorods obtained from ilmenite sand and the necessity of the metastable polymorphs formation was presented in 2008 and 2010 [132, 133]. It was revealed that for the formation of the nanorods, instead of other particle morphologies, the formation of the metastable phases like Ti_2_O_3_ to Ti_3_O_5_ is crucial. The ilmenite mineral was ball-milled with the presence of activated carbon at a ratio 4:1 at room temperature and under vacuum. The role of the latter as mechanical activation agent was to trigger the initial reduction to TiO_2_. The obtained ultra-fine powder was annealed first at different high temperatures (900–1200 °C) in order to form the metastable phases. The low and controlled heating rate (5–10 °C) in an argon atmosphere with hydrogen flow was a critical step in order to obtain the desired metastable phases. It was reported that the presence of nitrogen led to an alternative redox reaction and ﻿iron nitride was formed. At temperature less than 1100 °C, the formed phase was rutile, which could remain in the same form after the second annealing step. The optimum duration of annealing was 8 h at 1200 °C, since prolonged heat treatment led to the formation of FeTi alloys. The second step of annealing was conducted at 700 °C in a ﻿N_2_–5%H_2_ atmosphere. The result was the gradual formation of TiO_2_ nanorods and iron. As can be seen from the SEM images and XRD spectra in Fig. 20, the intermediate phase started to transform to nanorods after 4 h. The length of the nanorods was dependent on the time of annealing, and after 8 h, the intermediate phase was entirely transformed to rutile nanorods of the maximum length. Extension of the thermal treatment led to the sintering of the 1-D structure to coarse nanoparticles.

**Fig. 21:**
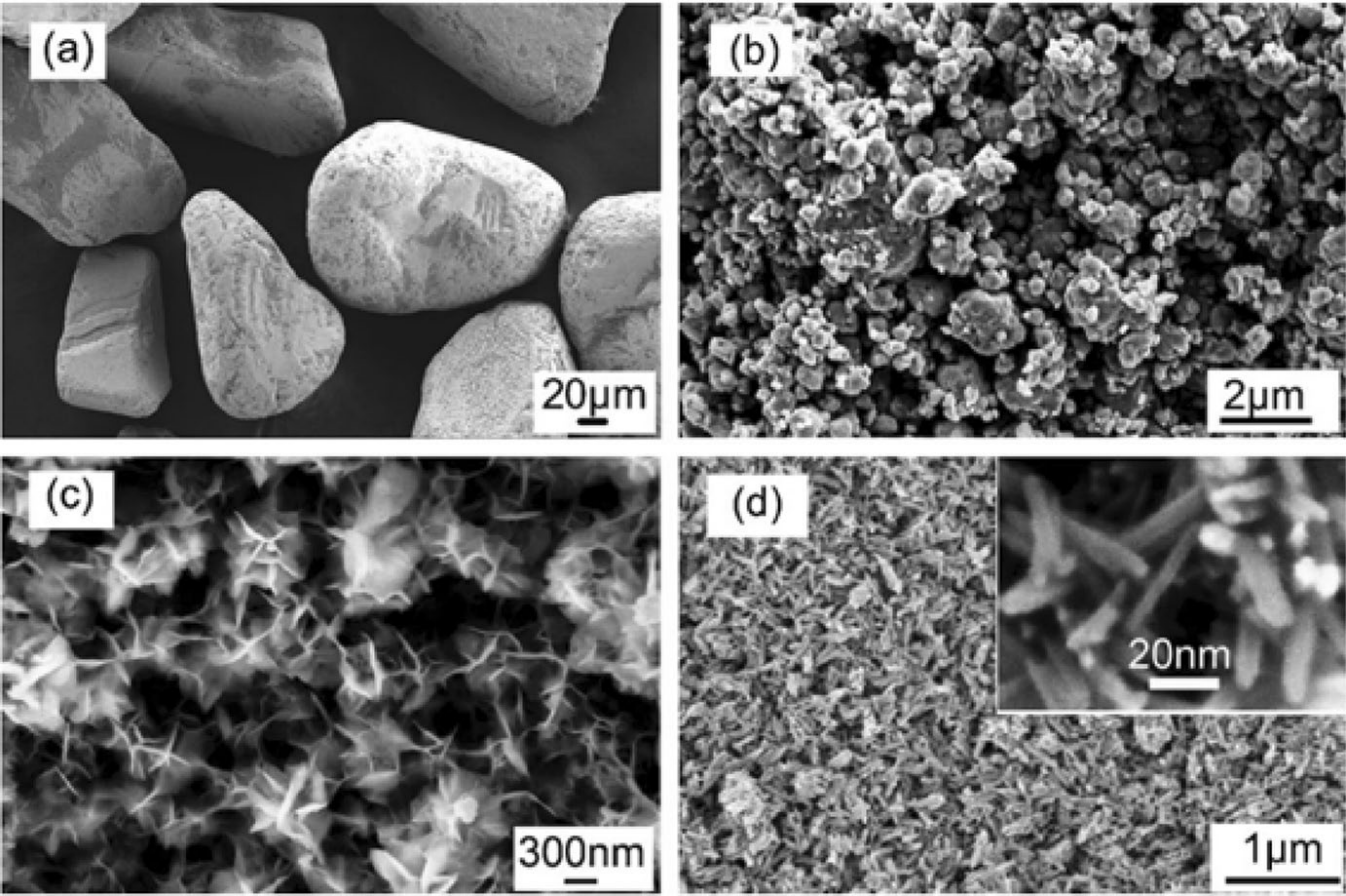
SEM images of original ilmenite powder (**a**), ball-milled ilmenite powder (**b**), flower-like FeTiO_3_ nano-structures after treatment with NaOH (**c**), and the obtained nanorods after treatment with HCl for 8 h (**d**); inset: a higher-magnification capture of the nanorods. Reprinted with permission from [134]. Copyright (2013) Wiley

﻿In 2013, Tao et al. [134] demonstrated a new method for the synthesis of TiO_2_ nanorods (single-crystal) from natural ilmenite. The BM pre-treated mineral was further wet-chemistry-treated by mixing in a 2 M NaOH aqueous solution for 2 h at 120 °C, and flower-like FeTiO_3_ nanoparticles were formed, but the authors concluded that this stage is an optional one. After short and mild drying, treatment with 4 M HCl at 90 °C for 4 h took place. The proposed mechanism was based on dissolution to TiOCl_2_ and FeCl_2_, hydrolysis, and precipitation. During the hydrolysis, TiO_2_ crystals started to precipitate and grow in a 1-D fashion. The finally obtained rutile TiO_2_ ﻿tetragonal nanorods (Fig. 21) had a length in the range of 50–100 nm, width of 5–20 nm, and thickness of 2–5 nm. The nanorods have also a moderately high specific area for this kind of nano-structure (up to 97 m^2^/g). The most interesting outcome was that they showed excellent photocatalytic capability towards the photodegradation of oxalic acid, analogous with the one of Sigma–Aldrich's Degussa P25.

**Fig. 22:**
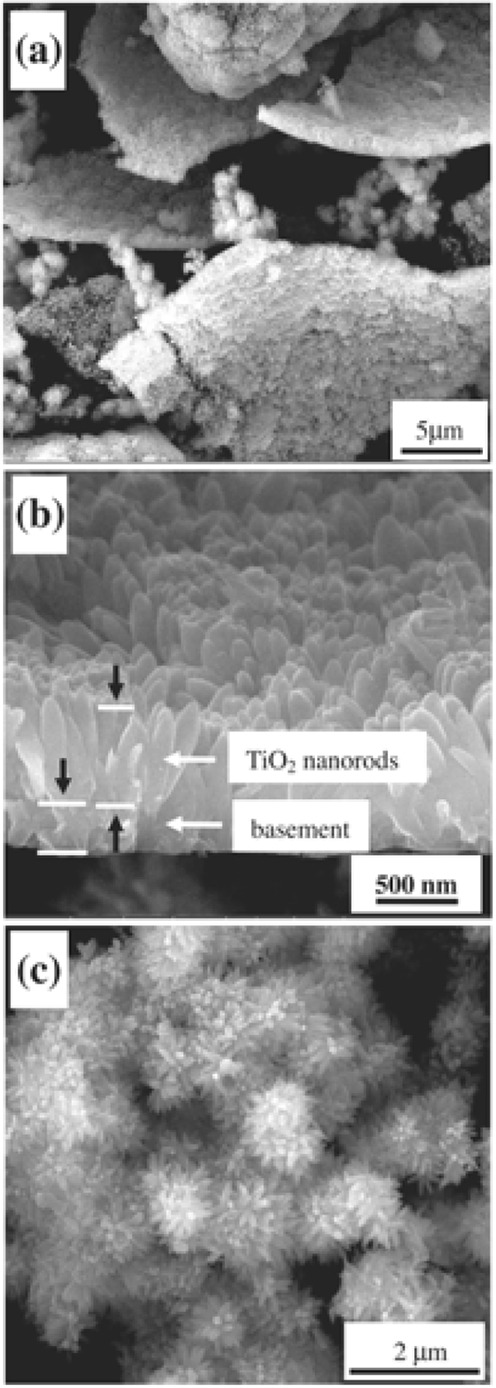
SEM images of ball-milling-derived amorphous Cu_50_Ti_50_ after immersing in HNO_3_ aqueous solution for 48 h. Reprinted with permission from [135]. Copyright (2014) Elsevier

In 2014, Zhao et al. [135] reported the formation of spindle-like rutile TiO_2_ nanorods from the dealloying in acidic conditions of an amorphous Cu_50_Ti_50_ alloy. The latter was formed by high-energy ball milling of elemental Cu and Ti in an argon atmosphere. In order to avoid the high temperature as a result of the BM process, after milling for 0.5 h, there was an interruption for also 0.5 h. The as-received material was immersed in a highly concentrated HNO_3_ aqueous solution (13.14 M) for dealloying. The obtained nanorods (Fig. 22) revealed a good photocatalytic degradation capability against the dye methyl orange under UV light irradiation, via the formation of radicals.

**Fig. 23:**
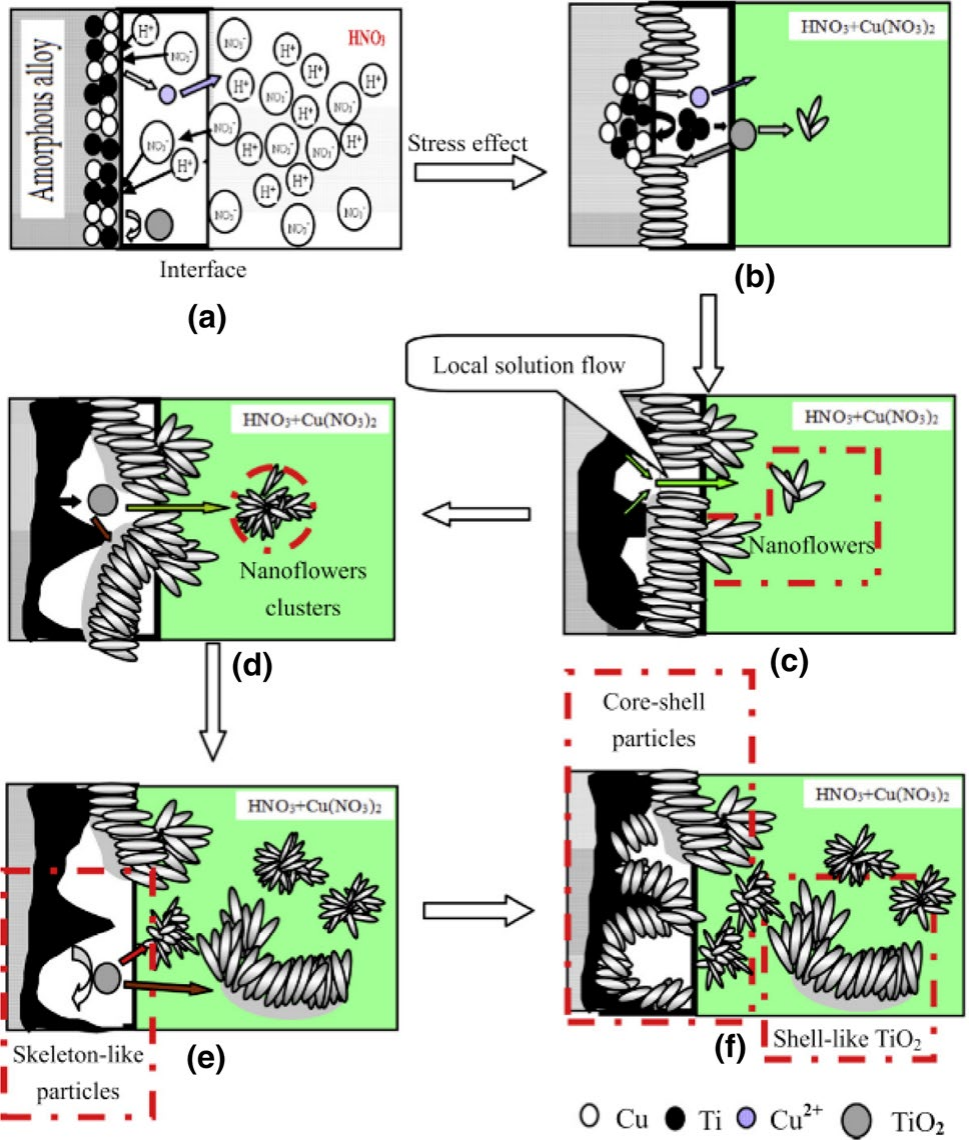
A schematic illustration of all the involved steps/mechanisms for the dealloying of amorphous Cu_50_Ti_50_ powders to rutile TiO_2_ nanorods. Reprinted with permission from [135]. Copyright (2014) Elsevier

In the work of Zhao et al. [135], the involved steps/mechanism for the formation of the TiO_2_ nanorods from the raw Cu_50_Ti_50_ alloy were proposed (Fig. 23). Titanium metal cannot react with nitric acid due to the presence of an oxide film. With the mechanical stress that is applied from the ball milling, the dealloying starts by corrosion from the outer surface and the removal of copper atoms and gradually continues to the inner part of the alloy.

## Conclusions—Prospectives

Taking into account all the above-mentioned results, it is reasonable to conclude that the formation of the hotspots as well the localized temperature increase during the mechanochemical treatment can antagonize the harsh conditions created inside the autoclave during the hydrothermal treatment, especially when the particle size of the precursor is equal or less than that of P25. It can be suggested that the thermal effects during the synthesis when the temperature is higher than 150 °C overcome the effects of the US pre-treatment. Especially in the case of a basic hydrothermal process, the utilization of sonication (US) as the pre-treatment has a vital role in the formation of 1-D nanostructures. The US effects can be further explored and applied for the synthesis of novel nano-engineered materials by other methods like precipitation, targeting towards the achievement of specific physical, chemical, and optical features.

In general, mechanochemistry can be a useful tool toward the manipulation of the important and desired features for different applications. Ball milling or US waves play a key role in size, shape, bandgap, porosity, light absorption, etc. Considering the above observations regarding the formation of NTBs, we can derive two possible conclusions/proposals. First, the presence of Fe stabilizes the layered structure of the nano-petals to roll to tubes. Second, the utilization of BM promotes the peeling of the mineral’s particle even at a lower concentration of 10 M, necessary for the hydrothermally peeling of TiO_2_ particles.

The herein presented results showed that the mechanochemical-derived forces can promote the features of the catalyst, crucial for heterogeneous photocatalytic applications. While the main goal of the research effort towards the formation of 1-D TiO_2_ up to nowadays was focused predominately on the explanation of the involved steps and mechanisms, in the cases where the materials were tested as photocatalyst, they revealed elevated photocatalytic capabilities, equal or better compared to the benchmark P25 in most of the cases. The goal of the present work is to highlight the developments in the area mechanochemical approaches when designing new synthetic strategies of nanostructured materials, as well as to call and initiate the attention for the possibilities for future utilization and exploration. We believe that nanoscaled and especially nanotubular-shaped titania can be further studied as photocatalyst, and we actively work towards this direction. Applying mechanochemistry will also be interesting to conduct for the design and synthesis of novel nanostructured electrodes for electrochemical catalytic reactions. Even though it is impossible these two techniques are simultaneously combined, the utilization of both at separate steps of synthesis can beget innovative approaches towards the synthesis of highly photo-active zero- and/or one-dimensional titanium-based catalyst, pure or doped with heteroatoms, like nitrogen or metals. In-depth study of the photocatalytic properties and applications of the TiO_2_ NTBs, as, for instance, advanced oxidation processes or biomass valorization, can lead to interesting and important outcomes, as occurred in the case of their application in electrocatalysis and photo-remediation. Additionally, the use of a simple and economic US bath or ball-milling grinder can be utilized as a powerful synthetic tool. It is also important to point out that the use of mechanochemical processes in lab during the synthesis can lead to effects not yet studied, hypothesized, or imagined. Last but not least, we would like to emphasize that it will be absolutely beneficial if more details are provided when mechanochemistry is applied, such as calorimetric evaluation of the setup, luminol mapping, experimental setup details (horn details, photos, or a drawing), and details of the synthesis (yield, purity, size separation techniques, etc.).
